# Inter‐microscope comparability of dental microwear texture data obtained from different optical profilometers: Part I Reproducibility of diet inference using different instruments

**DOI:** 10.1002/ar.25685

**Published:** 2025-05-10

**Authors:** Daniela E. Winkler, Mugino O. Kubo

**Affiliations:** ^1^ Department of Natural Environmental Studies The University of Tokyo, Graduate School of Frontier Sciences Kashiwa Chiba Japan; ^2^ Kiel University, Zoological Institute, Zoology and Functional Morphology of Vertebrates Kiel Schleswig‐Holstein Germany

**Keywords:** dental wear, diet reconstruction, DMTA, roughness, standardization, topography

## Abstract

Dental microwear texture analysis (DMTA) has become a well‐established method for dietary inference and reconstruction in both extant and extinct mammals and other tetrapods. As the volume of available data continues to grow, researchers could benefit from combining published data from various studies to perform meta‐analyses. However, the different optical profilometers used to capture three‐dimensional surface scans for DMTA are known to produce variation even when measuring the same surface. In this study, we compare DMTA data of 36 guinea pigs that received different diets in a controlled feeding experiment, measured using five different instruments: three confocal‐scanning microscopes and two confocal laser‐scanning microscopes. Each dataset is filtered according to in‐house standards of the respective laboratories. Our findings reveal inter‐microscope differences in the majority of the 40 DMTA parameters analyzed. Height and volume parameters were the most consistent across instruments, whereas density and complexity parameters exhibited pronounced differences. We thus propose DMTA parameters that were stable regardless of microscope. Despite these inter‐microscope variations, the overall results from all instruments consistently show the same dietary differentiation among the guinea pig feeding groups, supporting the suitability of DMTA for reproducible and objective dietary inferences. To enhance data exchange, inter‐lab comparability, and collaboration in the future, we propose a roadmap that includes the introduction of device‐specific correction equations.

## 1 INTRODUCTION

The analysis of microscopic ingesta‐induced wear features on teeth to infer dietary preferences of extant (e.g., Aiba et al., 2019; Calandra & Merceron, 2016; Schubert et al., 2010; Schulz et al., 2010; Schulz et al., 2013; Scott et al., 2012; Winkler, Schulz‐Kornas, Kaiser, De Cuyper, et al., 2019), extinct mammals (e.g., DeSantis, 2016; Kubo & Fujita, 2021; Scott et al., 2005; Ungar et al., 2007) and other non‐mammalian vertebrates (e.g., Bestwick et al., 2019; Kubo et al., 2023; Sakaki et al., 2022; Winkler, Iijima, et al., 2022; Winkler, Kubo, et al., 2022; Winkler, Schulz‐Kornas, Kaiser, & Tütken, 2019), as well as use‐wear analysis to infer the function of man‐made tools (Calandra, Pedergnana, Gneisinger, & Marreiros, 2019; Calandra, Schunk, Bob, et al., 2019; Calandra, Schunk, Rodriguez, et al., 2019), is of great interest to a broad community of biologists, paleontologists, and archeologists. Methods have undergone distinct modifications since (2D stereomicroscopic) microwear analysis was developed by Baker et al. (1959) and gained popularity since Walker et al. (1978). Recently, stereomicroscopic microwear analysis has been replaced by dental microwear texture analysis (DMTA), which is a semi‐automated, repeatable, and supposedly less observer‐biased approach than 2D microwear (Grine et al., 2002) that captures three‐dimensional surface data. Within DMTA, different algorithms are applied to quantify surface texture patterns according to standardized scale‐sensitive fractal analysis (SSFA) (e.g., Scott et al., 2005, 2012; Ungar et al., 2003, 2007) or International Organization for Standardization (ISO) surface roughness parameters (Schulz et al., 2010, 2013). The current market standard for parameter computation is the software MountainsMap (Digital Surf, Besançon, France), though recently a free, R‐implemented analysis tool has been published (Thiery et al., 2024). Additionally, MountainsMap‐specific surface parameters such as mean depth and density of furrows, mean height, mean area, or motif analysis have been found indicative of dietary preferences and are frequently included in DMTA studies (e.g., Schulz et al., 2013; Schulz‐Kornas et al., 2019; Winkler, Schulz‐Kornas, Kaiser, De Cuyper, et al., 2019; Winkler, Schulz‐Kornas, Kaiser, & Tütken, 2019; Winkler et al., 2020, 2021, 2022). However, the implementation of several SSFA parameters in a new module in MountainsMap has been found to produce divergent data compared to the original SSFA conducted in Sfrax/Toothfrax (Calandra et al., 2022).

DMTA is relatively accessible through various types of digital microscopes, including interferometry, confocal, and laser‐scanning microscopes of different price ranges. Different labs have established workflows based on their specific instruments, generating large amounts of data that could facilitate meta‐analyses if made available. However, data availability is currently a debated issue, not only within the DMTA community, but also across disciplines (Tedersoo et al., 2021). The majority of scientific journals encourage or mandate raw data to be made available through online repositories, yet two major problems persist:

1. Not all researchers agree to make raw data accessible at the time of publication. This could be due to lax journal data availability policies, cost of storage services such as Dryad (even though many free repositories like Zenodo exist), tedious processes for data repository description and upload processes (Marwick & Birch, 2018) or because they want exclusive access to their data for follow‐up studies.
2. Data is scattered across platforms, archives, and repositories (e.g., Zenodo, Dryad, github), complicating data corroboration.

A potential solution is a joint initiative to store raw DMTA data, similar to resources such as MorphoSource (www.morhposource.org) or the Paleobiology Database (paleobiodb.org). Achieving raw data availability raises another issue: the comparability of data obtained under different conditions. Data quality can potentially be influenced on three levels:

Firstly, whether scans are obtained directly from original specimens (teeth, bones, artifacts), casts, or molds. Mihlbachler et al. (2019) found that scans obtained from casts gave significantly different DMTA results compared to scans obtained from original enamel surfaces, and that discrimination between dietary groups was thus diminished.

Secondly, Goodall et al. (2015) highlighted not only differences between scans obtained from originals and casts, but also a second obstacle, that some molding silicones are more accurate than others. It is therefore crucial to know how data were obtained (from original versus mold/cast, which needs to be clearly indicated in the data repository descriptions), and to be aware of potential variation in fidelity of surface reproduction from different molding compounds. Still, such methodological problems can be easily tackled by extending comparative studies of the widely available molding materials (Goodall et al., 2015; Sawaura et al., 2022) and urging the community toward using the highest precision material available.

The third level of data quality differences, however, may potentially be the largest hindrance in making available data comparable. Data gathered from different confocal (blue and white light) profilers and laser‐scanning microscopes has been found to differ for the same specimens. Even within the same product line (five different Sensofar *PLμ* machines or two different Olympus LEXT machines), the same surface scan obtained on different microscopes produced different DMTA data (Arman et al., 2016, 2019). The authors found that inter‐microscope differences can be reduced using automated pre‐analysis filter routines. Similarly, for two confocal laser‐scanning microscopes of the same product line (Keyence VK), Kubo et al. (2017) found that the application of different pre‐analysis filter routines had a stronger effect on DMTA data than the microscope used. These are, up to now, the only studies comparing inter‐microscope differences when generating DMTA data from biological surfaces. Besides Sensofar, Olympus, and Keyence, confocal (laser and light) microscopes manufactured by Leica (DCM8), Zeiss (LSM 800) and NanoFocus (*μ*surf Custom) are frequently used to obtain DMTA data. The different scanning methods and specifications (numerical aperture, spatial and lateral resolution, etc.) will likely result in different results when scanning the same sample (Calandra, Schunk, Bob, et al., 2019), a problem that has often been discussed by metrologists (see Arman et al., 2016; Calandra, Schunk, Bob, et al., 2019; Jiménez‐Manchón et al., 2024 and references within). Moreover, manufacturers do not share how data is processed from capture to output, thus we cannot assume that data will be comparable when obtained on these different instruments. While streamlining post‐scanning filtering protocols (and statistical approaches for analysis) and using high‐precision molding materials can help, consistent filtering templates might not be applicable in all contexts, as they have been developed within each lab for the specific device.

Consequently, if we want to achieve comparability and make use of the massive amounts of data generated by the community in the future, we need to quantify inter‐microscope differences and establish protocols to increase comparability and reproducibility. We decided to address this issue through a two‐part study. In the present paper, we focus specifically on reproducibility—examining whether the differences observed between dietary groups can be consistently detected even when different instruments are used. The second paper (Kubo et al., 2026, this issue) places its main emphasis on developing methods for correcting inter‐microscope differences. In this paper, we compiled a dataset of 36 guinea pigs who received six homogenous diets in groups of six individuals during a 3‐week feeding experiment (Winkler et al., 2021; Winkler, Schulz‐Kornas, Kaiser, De Cuyper, et al., 2019), and scanned them on five instruments: a confocal (blue LED) light microscope (*μ*surf Custom), a 3D profiling microscope with combined focus variation, confocal, and phase‐shifting interferometry mode, used in confocal setting with a blue LED (Sensofar S neox), a 3D profiling microscope with combined confocal and phase‐shifting interferometry mode, used in confocal setting with a white LED (DCM8 Leica), and two confocal (violet) laser‐scanning microscopes (Keyence VK‐9700, Keyence VK‐X3000). All instruments are equipped with comparable 100× long‐distance objectives, though scanning fields differ. Spatial and vertical resolution and other specifications are similar between all instruments (Table 1). It should be noted that DCM8 has been based on a collaboration of Leica with Sensofar, and its basic components are identical to the Sensofar S neox. However, the used LED and objectives differ.

**TABLE 1:** Technical specifications of the five confocal microscopes employed in this study.

	Model				
	*μ*surf custom	Sensofar S neox	DCM8 Leica	Keyence VK‐9700	Keyence VK‐X3000
Scanning mode	Confocal microscopy	Confocal microscopy	Confocal microscopy	Confocal microscopy	Confocal microscopy
Vertical (*z*) resolution (μm), *z*‐step size	0.016	0.2	0.2	0.1	0.1
Light source	Blue LED (470 nm)	Blue LED (460 nm)	White LED	Violet laser (408 nm)	Violet laser (404 nm)
Objective	100 × L	100×	100×	100 × L	100 × L
Numerical aperture	0.8	0.9	0.9	0.73	0.73
CCD camera resolution	984 × 984 pixels	1232 × 1028 pixels	1088 × 776 pixels	1024 × 768 pixels	1024 × 768 pixels
Scan size (μm)	160 × 160	169 × 141	140 × 100	140 × 105	140 × 105
Spatial (*x*, *y*) resolution (μm)	0.16	0.14	0.129	0.137	0.137

We scanned the same dental molds from the 36 guinea pigs on all microscopes and compared results in terms of relative differences between diet groups, but also in terms of absolute parameter values. As already published data may not be available, or only as filtered data, we are aiming to enhance comparability for already published data. We hence processed data according to published protocols for each instrument and analyzed resulting differences. We then attempted applying correction factors from Kubo et al. (2026, this issue) for each instrument. Under the given conditions, scanned areas could not be perfectly matched across instruments, as the original scans were not taken with a repeated‐measurement design in mind (see Böhm et al., 2019 and Calandra, Schunk, Rodriguez, et al., 2019 for best practice how to prepare samples and the measuring system to match scans in a repeated‐measurement design). Therefore, we also tested how well dietary differences can be reproduced when re‐scanning the same individuals with slightly varying scanning positions.

## 2 MATERIALS AND METHODS

### 2.1 Diet groups

The data presented here is based on a controlled feeding experiment performed in 2017 and published in Winkler, Schulz‐Kornas, Kaiser, De Cuyper, et al. (2019). Guinea pigs received six specific diets in groups of six individuals over the course of 5 weeks. The diets were: Fresh‐cut alfalfa(lucerne) = LF, dried alfalfa(lucerne) hay = LD, fresh‐cut Timothy grass = GF, dried Timothy grass hay = GD, fresh‐cut bamboo leaves with small stems = BF, dried bamboo leaf hay = BD.

For detailed characterization of phytolith content and dry ash, see Winkler, Schulz‐Kornas, Kaiser, De Cuyper, et al. (2019).

### 2.2 Molding and data acquisition

Silicone molds of the upper right dentition for 36 guinea pigs were made using high‐resolution silicone (Provil novo Light C.D.2 fast set EN ISO 4823, type 3 light, Heraeus Kulzer GmbH, Dormagen, Germany). We decided to measure molds, because the jugular bone would contact the microscope's objective and obstruct the desired measuring position. The measurements were conducted by one person (DEW) and took place about 5 years apart, using the same silicone molds. They were first scanned in spring 2018 on a *μ*surf Custom confocal disc‐scanning microscope, at the Center of Natural History, University of Hamburg (now LIB Hamburg). Further details can be found in Winkler, Schulz‐Kornas, Kaiser, De Cuyper, et al. (2019); the original raw surface scans are published in Winkler et al. (2021).

The second time, the molds were scanned on a Keyence confocal laser‐scanning microscope VK‐9700, at the Graduate School of Frontier Sciences, the University of Tokyo, in spring 2021. In summer 2022, the same molds were then taken to the Palevoprim lab (CNRS) at the University of Poitiers, France, and scanned with the confocal DCM8 Leica 3D surface metrology microscope and the Leibniz institute for the Analysis of Biodiversity Change (LIB) Hamburg, Germany, where they were scanned using the confocal 3D profiling microscope Sensofar S neox. The final dataset was obtained in summer 2023, again at the Graduate School of Frontier Sciences, the University of Tokyo, using the confocal laser‐scanning microscope VK‐X3000. Technical specifications of all microscopes are given in Table 1.

### 2.3 Description of scanning and filtering procedure

**FIGURE 1:**
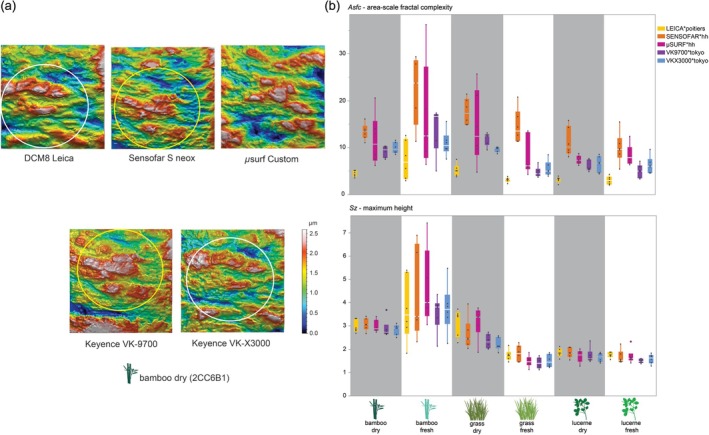
Comparison of data derived from all five microscopes. (a) Exemplary 2D views of the topographic height maps of surfaces from one individual of the bamboo dry group. Note that the scanning area does not perfectly match between microscopes, but that overlapping areas can be found (marked in white and yellow circles). Scans are to the same scale (μm). (b) Exemplary DMTA parameters that are hardly comparable (upper: *Asfc*) and well comparable (lower: *Sz*) between microscopes.

In all instances, the anterior most enamel band of the upper right fourth premolar was scanned, and up to four non‐overlapping scans were taken. We were unable to match the exact same position of the previous scans. However, as this enamel band in guinea pigs is narrow and short, it is very plausible that the re‐scanned areas have a huge overlap with the previously scanned areas. Following Winkler, Schulz‐Kornas, Kaiser, De Cuyper, et al. (2019), scans were then manually cropped in MountainsMap to 60 × 60 μm, because enamel bands in guinea pigs are generally smaller in width than the default scanning areas (e.g., *μ*surf Custom: 160 × 160 μm, Keyence VK‐9700: 140 × 105 μm). The positioning of individual cutouts will likely differ between scans obtained on the five instruments (Figure 1a). As an exact match cannot be ensured, the study also serves as a test of how well results can be reproduced when re‐scanning the same individuals and slightly varying the scanning positions.

Further data processing was conducted in MountainsMap Imaging Topography (version 9.0.9878 with “Grains & Particles,” “3D Advances Surface Texture,” and “Scale‐Sensitive Analysis” modules, DigitalSurf, France), including re‐analysis of the data obtained from the *μ*surf Custom at the University of Hamburg in 2018 and originally analyzed in MountainsMap version 7.4.8676 (Winkler, Schulz‐Kornas, Kaiser, De Cuyper, et al., 2019). Data were first treated according to the published filtering routines for each device:

1. Tokyo‐template: Applying a slightly modified version of the standard protocol established for the Keyence VK‐9700 at the Kubo lab (Aiba et al., 2019; Kubo & Fujita, 2021) which includes mirroring all surfaces in *x* and *z* (to compensate for the molding procedure), leveling (least‐square plane by subtraction), spatial filtering (robust Gaussian filter with a cutoff value of 0.8 μm), filling of non‐measured points using the smoothing function of Mountains Map, noise‐reduction by thresholding (upper and lower 0.5%), removal of outliers (maximum slope of 85°) and form removal (polynomial of increasing power = 2). This protocol will hereafter be referred to as *tokyo*.
2. Hamburg(hh)‐template: Following the standard protocol established for the *μ*surf Custom at the Kaiser Lab (Schulz et al., 2010, 2013) which includes mirroring all surfaces in *x* and *z* (to compensate for the molding procedure), leveling (least‐square plane by subtraction), spatial filtering (denoising median 5 × 5 filter size and Gaussian 3 × 3 filter size; default cutoffs are used), filling of non‐measured points using the smoothing function of Mountains Map, noise‐reduction by thresholding (upper and lower 0.5%), removal of outliers (maximum slope of 85°) and form removal (polynomial of increasing power = 2). This protocol will hereafter be referred to as *hh*.
3. Poitiers‐template: Following an updated standard protocol established at the Palevoprim lab (Merceron et al., 2016) which includes mirroring all surfaces in *x* and *z* (to compensate for the molding procedure), filling of non‐measured points by the mean of neighboring points, leveling (least‐square plane by subtraction), removal of aberrant peaks with automatic operators including a morphological filter (opening filter of 2 μm diameter, thresholding between 100 and 0.2 μm, subtraction of original and thresholded surface), leveling of the resulting difference surfaces, form removal (polynomial of increasing power = 2), spatial filtering (denoising median 5 × 5 filter size and Gaussian 3 × 3 filter size; default cut‐offs are used), and final leveling. This protocol will hereafter be referred to as *poitiers*.

The filtering protocols were applied for the data collected on the corresponding machine, resulting in five datasets:

1. LEICA*poitiers.
2. SENSOFAR*hh.
3. *μ*SURF*hh.
4. VK‐9700*tokyo.
5. VK‐X3000*tokyo.

Additionally, we applied the hard filter protocol suggested by Arman et al. (2016) to the raw data and included the results in the electronic supplement (Figure S2; Table S2). We computed a total of 40 surface texture parameters that are frequently applied to biological surfaces (Schulz et al., 2010, 2013; Scott et al., 2005; Ungar et al., 2003; Winkler et al., 2021; Winkler, Schulz‐Kornas, Kaiser, De Cuyper, et al., 2019; Winkler, Schulz‐Kornas, Kaiser, & Tütken, 2019), 30 of them stemming from the ISO 25178‐2 (2021). There are some differences from the parameter set published in Winkler, Schulz‐Kornas, Kaiser, De Cuyper, et al. (2019). We did not include the four ISO‐12871 flatness parameters, as they are directly derived from the ISO‐25178 height parameters *Sa*, *Sq*, *Sv*, and *Sz* and thus redundant. Similarly, we excluded the ISO‐25178 volume parameter *Vmp* because it is identical to *Vm* when using default cutoff settings. We further excluded the three main texture directions (*Tr1R*, *Tr2R*, *Tr3R*) and isotropy, but additionally included the SSFA parameters *Asfc* (area‐scale surface complexity), *HAsfc9* (heterogeneity of complexity for 3 × 3 cells) and *epLsar* (anisotropy) as these are among the most frequently applied measures of DMTA in other studies (Merceron et al., 2010; Schubert et al., 2010; Scott et al., 2005, 2012; Ungar et al., 2003, 2007). As the new SSFA implementation has two different versions of *epLsar* (“*new EpLsar*” and “*Sfrax epLsar*”), we are computing both.

### 2.4 Statistics

All statistical analyses were carried out in JMP Pro v.17 except for a Bonferroni correction, which was manually done on the compiled result in an Excel sheet. For each specimen, median values per parameter were calculated from up to 4 (at least 3) non‐overlapping scans (compare Winkler, Schulz‐Kornas, Kaiser, De Cuyper, et al., 2019). Because of the repeated‐measurement design, that is, the same specimens were analyzed five times, we performed a *t*‐test for paired samples and accounted for increased probability of Type I error due to multiple comparisons with a Bonferroni correction. We are including both raw *p*‐values and adjusted *p*‐values into the supplements (Table S1). Scripts for the statistical analyses executed in JMP are included in the Supporting Information.

## 3 RESULTS

### 3.1 Instrument‐specific filtering protocols

The absolute parameter values varied across the five microscopes; however, we observed consistent relative dietary differences between the diet groups on all instruments (Figures 1b and S1). The groups lucerne fresh (LF), lucerne dry (LD) and grass fresh (GF) showed similar values for most parameters (Figure S1; Table S2). The grass dry (GD) group exhibited values that were intermediate between these three groups and the two bamboo groups. The bamboo fresh (BF) and bamboo dry (BD) groups were similar to each other. Notably, BF often displayed greater variation compared to all other diet groups. Depending on the specific parameter, the pattern was hence either LF = LD = GF < GD < BF = BD or LF = LD = GF > GD > BF = BD. This consistent spacing between diet groups was observed across all datasets and for each microscope.

Paired *t*‐tests revealed that some microscopes were more comparable than others (Table S1). The fewest significant differences between datasets were obtained between the two Keyence laser‐scanning microscopes VK‐9700 and VK‐X3000, with only 4 out of 40 parameters showing significant differences. This was followed by the comparison between VK‐X3000 and the *μ*surf Custom (15 out of 40 parameters) and between VK‐X3000 and the Sensofar S neox (16 out of 40 parameters) (Tables 2 and S1).

**TABLE 2:** Inter‐microscope comparability for 40 DMTA parameters using the guinea pig natural diet dataset. Data from t‐test for paired samples with Bonferroni correction.

Machine A	Machine B	*n* sign. differences	*n* sign. differences corrected
VKX3000*tokyo	VK9700*tokyo	18	4
VKX3000*tokyo	*μ*surf*hh	28	15
*μ*surf*hh	Sensofar*hh	26	16
VK9700*tokyo	*μ*surf*hh	25	20
VKX3000*tokyo	sensofar*hh	31	22
VK9700*tokyo	Leica*poitiers	28	26
VK9700*tokyo	Sensofar*hh	34	30
VKX3000*tokyo	Leica*poitiers	35	32
*μ*surf*hh	Leica*poitiers	35	33
Sensofar*hh	Leica*poitiers	33	29

The BF group showed greater variability in volume, height, peak sharpness, density, and complexity parameters compared to other diet groups. On the two Keyence laser‐scanning microscopes (VK‐9700, VK‐X3000), the range of values for BF individuals was generally lower than those obtained with the other confocal microscopes. The Leica device (DCM8) produced significantly larger area parameters, dale (*Sdv*) and hill volume (*Shv*), and lower density of peaks (*Spd*) and furrows (*medf*) than the other microscopes (Figure S1). The *μ*surf Custom also showed lower density of peaks (*Spd*) and furrows (*medf*) than both Keyence instruments and the Sensofar S neox. The Sensofar S neox displayed significantly greater complexity parameters (*Asfc*, *nMotif*, *Sdr*) than the other instruments.

### 3.2 Individual parameter groups

#### 3.2.1 Area parameters

The diet groups LF, LD, and GF were characterized by low area parameter values, followed by GD; while the largest values were observed in BF and BD. Except for the DCM8 Leica, area parameter values for LF, LD, and GF were very similar across all microscopes, and for *Sda* this similarity extended to GD as well (Figure S1). For *Sha* and *mea*, the *μ*surf Custom showed higher parameter values than the two Keyence microscopes and the Sensofar S neox, yet still much lower than those values recorded from DCM8 Leica. For both BF and BD, parameter values differed between microscopes, but only the DCM8 Leica fell distinctly outside the range of the other instruments.

#### 3.2.2 Complexity parameters

Among complexity parameters, *HAsfc9* was best comparable between microscopes. *Asfc*, *Sdr*, and *nMotif* showed strong differences between instruments, with a distinct pattern that was consistent between parameters (Figures 1b and S1). The Sensofar S neox resulted in larger values than other microscopes, followed by the *μ*surf Custom for *Asfc* and *Sdr*. The DCM8 Leica always produced the lowest complexity parameter values.

For *Sdr* and *Asfc*, both lucerne groups and the GF group had the lowest values. GD and both bamboo groups showed higher values than the previous three groups. For *nMotif*, both lucerne and both grass groups showed larger values, while both bamboo groups were characterized by lower values.

#### 3.2.3 Density parameters

Autocorrelation length (*Sal*) was equally low for LF, LD, and GF, intermediate for GD, and highest in both bamboo groups (BD, BF). This trend was consistent between all microscopes (Figure S1). For LF, LD, and GF, the Sensofar S neox showed lower values than the other microscopes, while for the *μ*surf Custom GD had overall higher values. For *Spd*, the trend between diet groups was opposite, with LF, LD, GF, and partially GD showing a larger density of peaks than the two bamboo groups. Here, the DCM8 Leica and *μ*surf Custom were consistently showing lower values than the other microscopes for all diet groups. For *medf*, again the DCM8 Leica and *μ*surf Custom showed lower values than all other instruments.

#### 3.2.4 Direction parameters

The relative spacing between diet groups (LF, LD, GF, GD, BF, BD) was generally consistent for anisotropy (*New EpLsar*, *Sfrax epLsar*), while texture aspect ratio (*Str*) and texture direction (*Std*) were less comparable (Figure S1). The BD group had lower *Str* and higher anisotropy values compared to all other diet groups. GD had the greatest *Str* and lowest anisotropy values, while the other diet groups (BF, DF, LD, LF) showed intermediate values. The Leica DCM8 recorded higher values than the other microscopes for *Str* and *Sfrax epLsar* values. Anisotropy was generally lower when measured on Keyence VK‐9700, VK‐X3000, and *μ*surf Custom, and higher for samples measured on Leica DCM8 and Sensofar S Neox.

#### 3.2.5 Height parameters

For all height parameters, the relative differences between diet groups were generally consistent across the different microscopes (Figures 1b and S1). This indicates that while absolute values varied, the ability to differentiate between dietary groups remained robust. The BF diet group consistently showed higher values and greater variability across many height parameters. For the GF, LD, and LF groups, lower and more consistent values across all height parameters were found, regardless of the microscope used.

The Leica DCM8 and Sensofar S Neox frequently exhibited higher variability and typically reported higher values for maximum (*s10z*, *Sp*, Sz, *matf*) and mean height (e.g., *Sa*, *Sq*, *metf, meh*) measurements. In contrast, the VK‐9700 and VK‐X3000 tended to show lower or more conservative values. The *μ*surf Custom often occupied a middle ground, displaying moderate variability and values, with the exception of *Sp*, where strong variability was observed in the *μ*surf Custom and Sensofar S Neox for the BF group.

#### 3.2.6 Peak sharpness

The only peak sharpness parameter (*Spc*) was consistently larger in the BF group and lowest in the GF, LD, and LF groups (Figure S1). Data from *μ*surf Custom showed very high variability in the BF and GD groups, while the other microscopes recorded less variability.

#### 3.2.7 Plateau size parameters

The spacing between diet groups was consistent across all microscopes, with BD and BF showing the greatest *Smc* values, GD intermediate values, and GF, LD, and LF equally low values. For *Smr*, this trend is opposite, with GF, LD, and LF exhibiting the greater values, followed by GD, and BF and BD having the lowest values. For *Smr*, however, the Leica DCM8 produced much higher values than the other instruments for BF and GD, and the Sensofar S Neox showed greater variability.

#### 3.2.8 Slope

The Leica DCM8 showed consistently lower values compared to other microscopes (Figure S1). Sensofar S Neox and *μ*surf Custom exhibited higher values with moderate variability in the former and greater variability in the latter device for the diet groups BF and GD. Still, the spacing between diet groups was consistent on all instruments, with BD, BF, and GD showing greater slope values than GF, LD, and LF.

#### 3.2.9 Volume

For *Sdv* and *Shv*, the Leica DCM8 produced the greatest variability among all microscopes, and also the highest values for each diet group. In contrast, for most other volume parameters, the Leica DCM8 showed the lowest values per diet group as compared to the other microscopes. Still, spacing between diet groups was consistent for all volume parameters, with LF, LD, and GF showing the lowest values, followed by GD, and both bamboo groups showing the greatest parameter values (Figure S1).

#### 3.2.10 Potential corrections of published data

**FIGURE 2:**
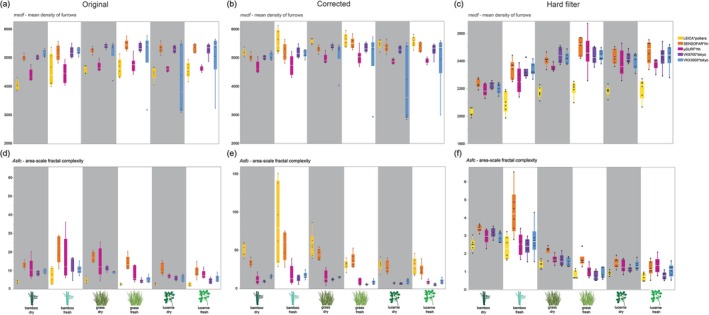
Comparison of results for the DMTA parameter *medf* and *Asfc*. (a) Original data for *medf* captured on the five different microscopes and filtered according to their published protocols. (b) Data for *medf* from all microscopes, filtered according to their published protocols and corrected using linear regression equations from Kubo et al. (2026, this issue) and compared to Keyence VK‐9700. (c) Data for *medf* from all microscopes filtered according to the hard filter protocol from Arman et al. (2016). (d) Original data for *Asfc* captured on the five different microscopes and filtered according to their published protocols. (e) Data for *Asfc* from all microscopes, filtered according to their published protocols and corrected using linear regression equations from Kubo et al. (2026, this issue) and compared to Keyence VK‐9700. (f) Data for *Asfc* from all microscopes filtered according to the hard filter protocol from Arman et al. (2016).

**FIGURE 3:**
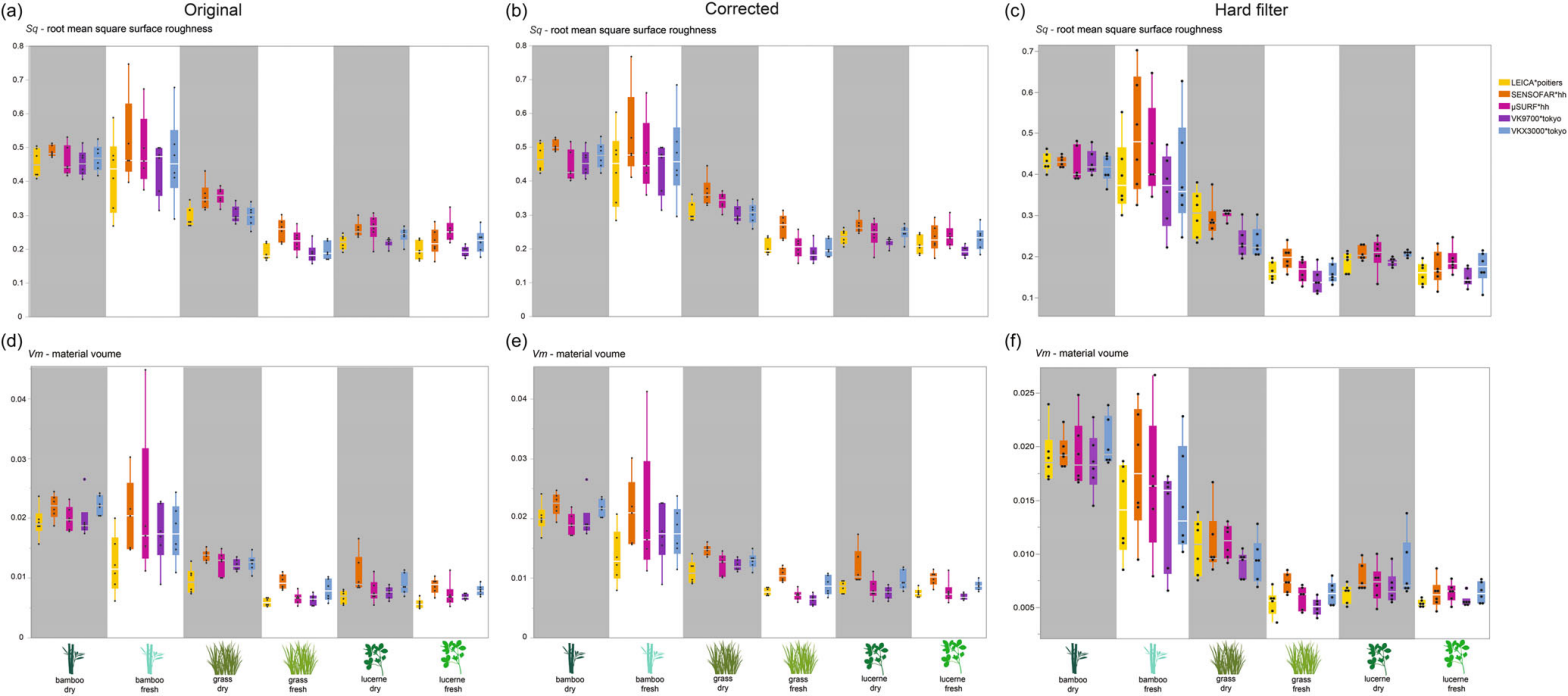
Comparison of results for the DMTA parameter *Sq* and *Vm*. (a) Original data for *Sq* captured on the five different microscopes and filtered according to their published protocols. (b) Data for *Sq* from all microscopes, filtered according to their published protocols and corrected using linear regression equations from Kubo et al. (2026, this issue) and compared to *Keyence VK‐9700*. (c) Data for *Sq* from all microscopes filtered according to the hard filter protocol from Arman et al. (2016). (d) Original data for *Vm* captured on the five different microscopes and filtered according to their published protocols. (e) Data for *Vm* from all microscopes, filtered according to their published protocols and corrected using linear regression equations from Kubo et al. (2026, this issue) and compared to *Keyence VK‐9700*. (f) Data for *Vm* from all microscopes filtered according to the hard filter protocol from Arman et al. (2016).

Kubo et al. (2026, this issue) suggested an approach by introducing a corrected factor for each instrument after re‐scanning standardized surfaces and matching the scanned areas on all instruments. By performing linear regression, it is thus possible to obtain such a correction factor for each individual DMTA parameter, and thus facilitate comparison between parameter values derived from datasets that were obtained on different instruments and treated with different post‐processing procedures. We are here comparing the results of such a correction based on the equations from Kubo et al. (2026, this issue) with the hard filter protocol by Arman et al. (2016) and the original data, filtered according to each instrument's standard protocol exemplarily, for the parameters *medf* (mean density of furrows), *Asfc* (area‐scale fractal complexity) (Figure 2), *Sq* (root mean square surface roughness), and *Vm* (material volume) (Figure 3). In this example, data obtained on Keyence VK‐9700 has been used as the baseline, resulting in the following correction equations for each microscope:

$${\text{medf}}_{\mathrm{VK}-9700}=2303.15479+{{\text{medf}}_{\text{Leica}}}^{*}\;0.71456,$$

$${\text{medf}}_{\mathrm{VK}-9700}=242.78428+{{\text{medf}}_{\text{μsurf}}}^{*}\;1.00846,$$

$${\text{medf}}_{\mathrm{VK}-9700}=-460.77763+{{\text{medf}}_{\text{Sensofar}}}^{*}\;1.09694,$$

$${\text{medf}}_{\mathrm{VK}-9700}=-499.35517+{{\text{medf}}_{\mathrm{VK}-X3000}}^{*}\;1.08042,$$

$${\text{Asfc}}_{\mathrm{VK}-9700}=-9.17786+{{\text{Asfc}}_{\text{Leica}}}^{*}\;12.678237,$$

$${\text{Asfc}}_{\mathrm{VK}-9700}=-1.65920+{{\text{Asfc}}_{\text{μsurf}}}^{*}\;1.13005,$$

$${\text{Asfc}}_{\mathrm{VK}-9700}=-1.95559+{{\text{Asfc}}_{\text{Sensofar}}}^{*}\;2.62701,$$

$${\text{Asfc}}_{\mathrm{VK}-9700}=-1.81005+{{\text{Asfc}}_{\mathrm{VK}-X3000}}^{*}\;1.71532,$$

$${\mathrm{Sq}}_{\mathrm{VK}-9700}=0.01531+{{\mathrm{Sq}}_{\text{Leica}}}^{*}\;0.99945,$$

$${\mathrm{Sq}}_{\mathrm{VK}-9700}=-0.02099+{{\mathrm{Sq}}_{\text{μsurf}}}^{*}\;1.01258,$$

$${\mathrm{Sq}}_{\mathrm{VK}-9700}=0.00529+{{\mathrm{Sq}}_{\text{Sensofar}}}^{*}\;1.02129,$$

$${\mathrm{Sq}}_{\mathrm{VK}-9700}=0.00642+{{\mathrm{Sq}}_{\mathrm{VK}-X3000}}^{*}\;1.00013,$$

$${\mathrm{Vm}}_{\mathrm{VK}-9700}=0.00227+{{\mathrm{Vm}}_{\text{Leica}}}^{*}\;0.92238,$$

$${\mathrm{Vm}}_{\mathrm{VK}-9700}=0.00121+{{\mathrm{Vm}}_{\text{μsurf}}}^{*}\;0.89375,$$

$${\mathrm{Vm}}_{\mathrm{VK}-9700}=0.00181+{{\mathrm{Vm}}_{\text{Sensofar}}}^{*}\;0.93683,$$

$${\mathrm{Vm}}_{\mathrm{VK}-9700}=0.00143+{{\mathrm{Vm}}_{\mathrm{VK}-X3000}}^{*}\;0.91743.$$

It has to be noted that Kubo et al. (2026, this issue) found *Asfc* could not be reliably corrected for the Leica DCM8 and the *μ*surf Custom. Applying the correction equation, it becomes apparent that discrepancies between instruments can be decreased but still exist for *Asfc* and *medf*. For the well‐comparable height and volume parameters *Sq* and *Vm*, the application of the correction equations does not substantially change the data. The hard filter template by Arman et al. (2016) strongly affects absolute parameter values but also makes data from the different instruments more similar (Table S2; Figures 2, 3, and S2).

## 4 DISCUSSION

### 4.1 Inter‐microscope differences

Not all parameters are suitable for direct comparison of absolute parameter values when data is obtained on different instruments. Our results clearly show that peak and furrow density‐related parameters are significantly different and should not be used for immediate comparison (Table S1; Figure S1). The source of this strong variation is likely due to the different scanning techniques and peak‐detection algorithms of confocal versus laser‐scanning microscopes. Numerical aperture also varied between 0.73 and 0.9 between the employed objectives, adding another source of variability. Application of different filtering protocols by each lab will likely further pronounce such data‐inherent differences. Therefore, for published and already filtered data (when raw data is not available), this comparability can be partially improved (compare Figure 2 *medf*) through the introduction of an instrument‐specific correction factor. However, several DMTA parameters were similar in absolute values between instruments and could be directly compared with higher confidence (Table 3). Comparability for these parameters can be further improved by applying the published correction equations, with some limitations for parameters that cannot be corrected, such as *Asfc* (Kubo et al., 2026).

**TABLE 3:** Comparison of DMTA parameters with <5 significant differences between (based on *t*‐test) and source of difference.

Parameter	Source of difference A	Source of difference B
*Sku*	Leica*poitiers	VKX3000*tokyo, Sensofar*hh, *μ*surf*hh, VK9700*tokyo
*Sz*	VKX3000*tokyo	*μ*surf*hh
	VK9700*tokyo	Leica*poitiers
*Std*	*μ*surf*hh	Leica*poitiers, VKX3000*tokyo, Sensofar*hh
*Vvv*	*μ*surf*hh	VKX3000*tokyo, VK9700*tokyo, Leica*poitiers
	VK9700*tokyo	Sensofar*hh
*S10z*	Sensofar*hh	VK9700*tokyo
*Sda*	Leica*poitiers	VKX3000*tokyo, VK9700*tokyo, Sensofar*hh, *μ*surf*hh
*Sdv*	Leica*poitiers	VKX3000*tokyo, VK9700*tokyo, Sensofar*hh, *μ*surf*hh
*matf*	Sensofar*hh	VKX3000*tokyo, VK9700*tokyo
	VK9700*tokyo	*μ*surf*hh
*HAsfc9*	Sensofar*hh	VK9700*tokyo

### 4.2 Comparability of results

The dietary differences between experimentally fed guinea pigs originally described by Winkler, Schulz‐Kornas, Kaiser, De Cuyper, et al. (2019) were confirmed for the newly obtained data from all microscopes. Both lucerne diets and the fresh grass diet were very similar in complexity, height, volume, area, and slope parameters. Especially low parameter values for height, volume, and the complexity parameters *Sdr* and *Asfc* are interpreted as related to low abrasive feeds (Kubo et al., 2017; Kubo & Fujita, 2021; Schulz et al., 2010, 2013; Winkler et al., 2020; Winkler, Schulz‐Kornas, Kaiser, De Cuyper, et al., 2019; Winkler, Schulz‐Kornas, Kaiser, & Tütken, 2019). On the contrary, higher parameter values would indicate a more abrasive diet. Even though slightly different enamel areas were scanned, as matching of the original areas was not possible, the re‐analysis gave the same result of increasing abrasiveness of experimental feeds in the order: lucerne fresh, lucerne dry, grass fresh < grass dry < bamboo fresh, bamboo dry (Figures 1b and S1). This shows that the previously derived interpretation of Winkler, Schulz‐Kornas, Kaiser, De Cuyper, et al. (2019) holds true; there is a significant effect of both silica content and hydration state of the plant tissue on the observed microwear texture pattern, with dry and more siliceous diets resulting in more abrasion. It also indicates that along the enamel facets of guinea pigs, there is little inter‐facet variation, which is also indicated in the observed low *HAsfc9* values (Figure S1). Therefore, our study also provides a test for reproducibility and reliability of DMTA results and strengthens the robustness of the method.

### 4.3 Outlook and suggested best practice

By finding the most stable (i.e., most comparable) parameters between the five microscopes, this study might help in the identification of 3D microwear texture parameters to focus on, as the huge availability has caused confusion and made it difficult to agree on a set of relevant parameters. By choosing the most stable ones, this controversy can be advanced. Nevertheless, stability is not the only relevant aspect; we still need to keep in mind that the selected parameters also should have high discriminatory power between diet groups. At least for comparative studies, where data is gathered in different labs and on different machines, we suggest concentrating on the parameters listed in Table 3. The height parameters *Sku, Sz, S10z*, and *matf*, the volume parameter *Vvv*, the complexity parameter *HAsfc9*, as well as *Sda*, *Sdv*, and *Std* were only significantly different between one to four combinations of microscopes. For *Sku*, *Sda*, and *Sdv*, the differences were caused by the Leica DCM8, indicating again that it is least comparable with the other instruments. Additionally, it may be advisable to consider subjecting the raw data (if available) to a strong filtering routine as proposed by Arman et al. (2016). Thus, it needs to be kept in mind that will result in a change in order of magnitude for each parameter (compare Figures 2 and 3).

In the current study, the source of difference between datasets was mostly the DCM8 Leica or the Sensofar S neox. The least comparable parameters were *Spd, nMotif, mea*, *and metf* (with 8 or 9 significantly different pairings). Therefore, these parameters should be excluded from comparative analyses. Among the other available parameters, many showed five to seven significantly different pairings between instruments. Still, as those parameters showed a consistent shift between diet groups (only differing in absolute value, but not in relative differences between diet groups), a correction factor could be used as proposed by Kubo et al. (2026, this issue) if one desires to include them in a comparative study.

Kubo et al. (2026, this issue), who compared standardized tooth samples and re‐scanned matching enamel areas on the same five microscopes employed in this study, showed how data captured on different microscopes can be integrated into a meta‐analysis by employing the height parameter *Sq* (RMS surface roughness) after applying a correction factor. Nevertheless, it must be noted that even after correction, results obtained from different microscopes need to be discussed as such, and the possibility of persisting inter‐microscope differences needs to be taken into account.

Such comparative studies should be conducted between more DMTA labs to understand specific characteristics of each microscope used, and to find best‐practice filtering protocols that facilitate inter‐microscope (and thus inter‐lab) data comparability. This will lead to a massive increase in comparative data available for future studies and avoid unnecessary data recollection. We highly encourage striving for a shared data repository to which DMTA research labs worldwide can contribute. As an initiative to promote such comparability, we propose to extend the approach of Kubo et al. (2026, this issue) to compile a standard set of molds from different typical specimens (e.g., ungulate, reptile, carnivore) and a few standardized flat surfaces (e.g., polished enamel) that are mounted on a microtiter plate with incision marks that can be aligned within a microscope‐specific coordinate system. Such custom standard samples shall be exchanged between DMTA labs, with each research group re‐scanning the same areas and processing the data according to their own preferred pre‐analysis protocol and the published protocols of other researchers. Subsequently, this data can be used to obtain accepted “correction equations” for each device, so that data can be shared and used between labs.

## 5 CONCLUSION

Reproducibility and less observer‐biased interpretation of results are two key advantages often cited when comparing DMTA to classical microwear analysis. Our study supports these claims, as data gathered from three different laboratories, on five different microscopes, and 4 years apart resulted in the same dietary discrimination between experimentally fed guinea pigs.

This study also highlights that, without correction, inter‐microscope comparison of published, filtered data can only be done for a few DMTA parameters. The majority of often applied parameters show significant inter‐microscope differences and require correction through a correction factor. Such correction factors could be obtained through a joint community effort which includes scanning of the same surfaces in multiple labs, which we propose here to our colleagues. Through our collaboration, we might achieve data comparability and advance research in our field.

## AUTHOR CONTRIBUTIONS

**Daniela E. Winkler:** Conceptualization; data curation; formal analysis; funding acquisition; investigation; methodology; visualization; writing – original draft; writing – review and editing. **Mugino O. Kubo:** Conceptualization; formal analysis; funding acquisition; methodology; project administration; resources; writing – review and editing.

## CONFLICT OF INTEREST STATEMENT

The authors declare no conflicts of interest.

## Supporting information


Data S1.

Table S1.

Table S2.

## Data Availability

All original, filtered surface texture scans used in this study are available online under https://doi.org/10.57892/100-71. Current reviewer version: https://opendata.uni‐kiel.de/receive/fdr_mods_00000071?accesskey=Ytu3RXfVUQ8EUuquEjnFyOxfO8h0ObmC. Unfiltered data from Winkler, Schulz‐Kornas, Kaiser, De Cuyper, et al. (2019); Winkler et al. (2021) has been published under https://doi.org/10.25592/uhhfdm.9163.
