# Supplementary material for: Inter‐microscope comparability of dental microwear texture data obtained from different optical profilometers: Part I Reproducibility of diet inference using different instruments
Source: Anat Rec (Hoboken). 2025 May 10;309(9):2442–54. doi: 10.1002/ar.25685 (PMC13431920; doi:10.1002/ar.25685)
Supplement: Supplementary file 1 — Data S1. [file AR-309-2442-s001.docx]

**Electronic Supplement for**

**Inter-microscope comparability of dental microwear texture data obtained from different optical profilometers: Part I Reproducibility of diet inference using different instruments**

Daniela E. Winkler^1,2,*^ & Mugino O. Kubo^1^

^1^Department of Natural Environmental Studies, The University of Tokyo, Graduate School of Frontier Sciences, Kashiwa, Chiba, Japan

*corresponding author: [dwinkler@zoologie.uni-kiel.de](mailto:dwinkler@zoologie.uni-kiel.de)

https://orcid.org/0000-0001-7501-2506

[mugino@k.u-tokyo.ac.jp](mailto:mugino@k.u-tokyo.ac.jp)

https://orcid.org/0000-0002-7748-7377

^2^Kiel University, Zoological Institute, Zoology and Functional Morphology of Vertebrates, Kiel, Schleswig-Holstein, Germany

This supplement contains:

Figure S1-S2, Table S3, JMP script for paired t-test

Tables S1 and S2 can be found as two separate Excel worksheets

| **Area** | |
| --- | --- |
| *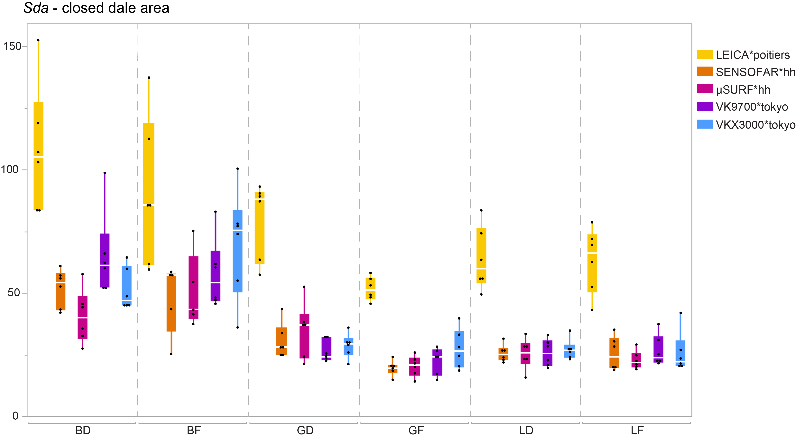* | *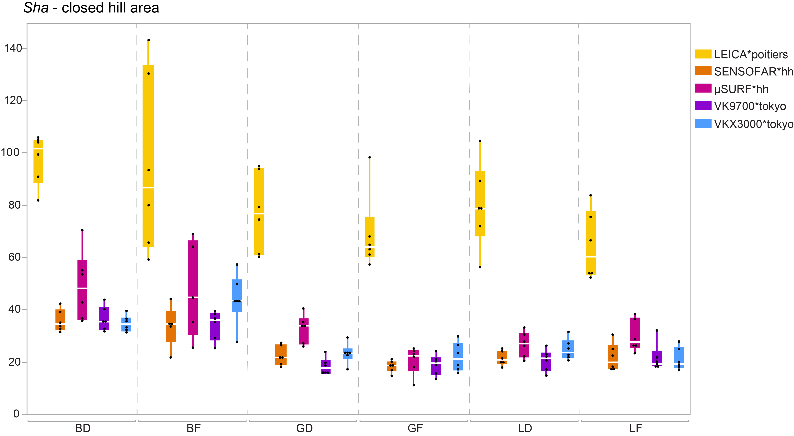* |
| *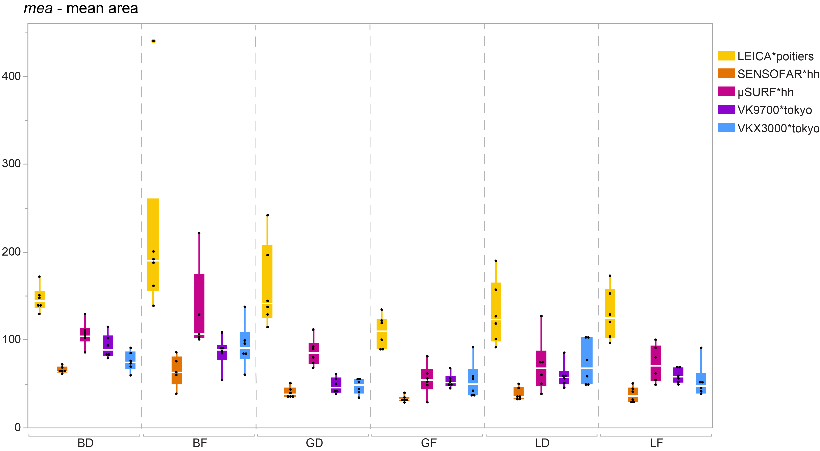* | |

| **Complexity** | | |
| --- | --- | --- |
| *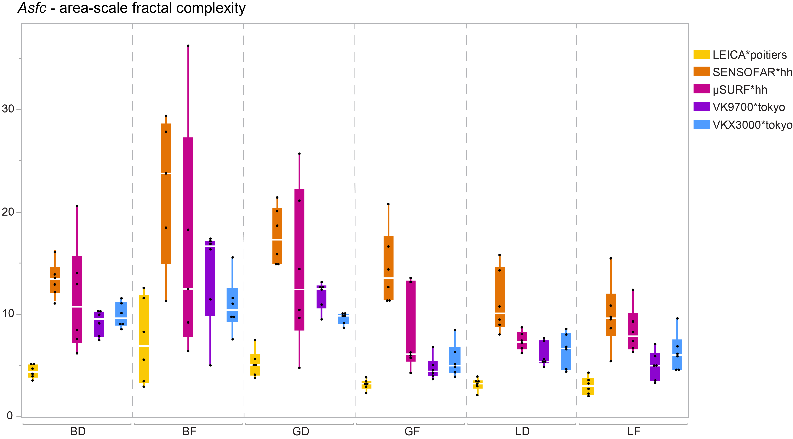* | *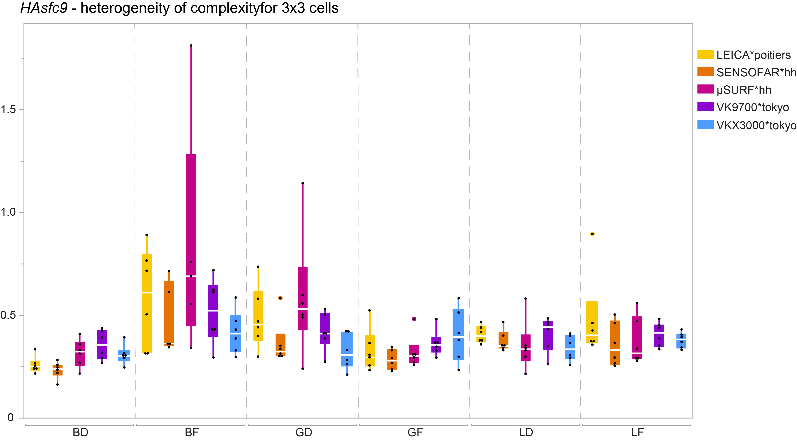* | *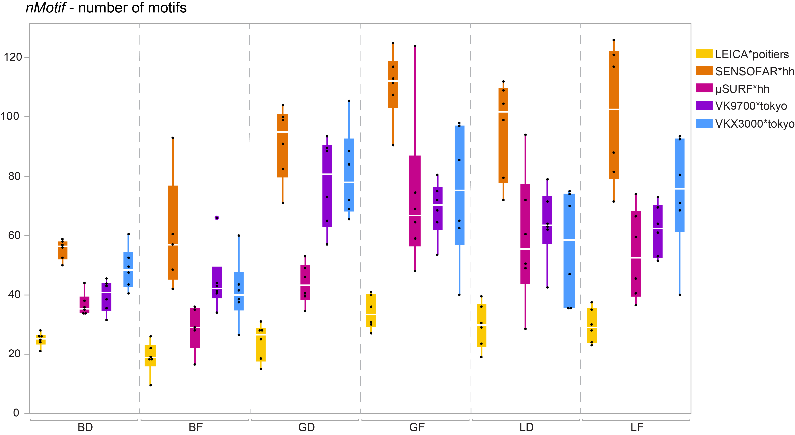* |
| *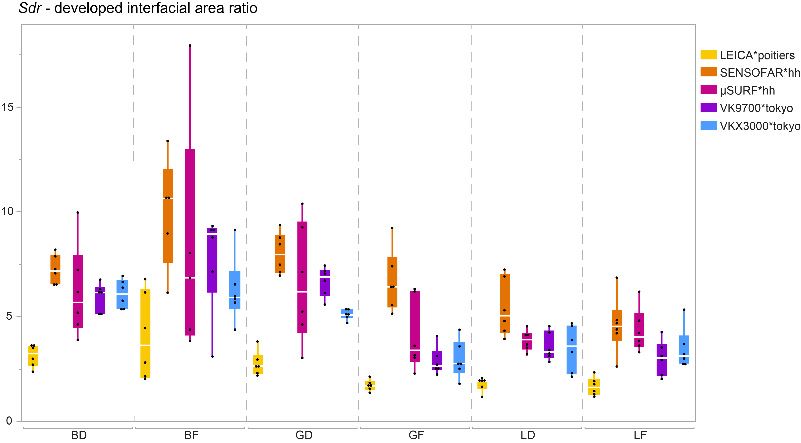* | | |

| **Density** | | | | | | | | |
| --- | --- | --- | --- | --- | --- | --- | --- | --- |
| *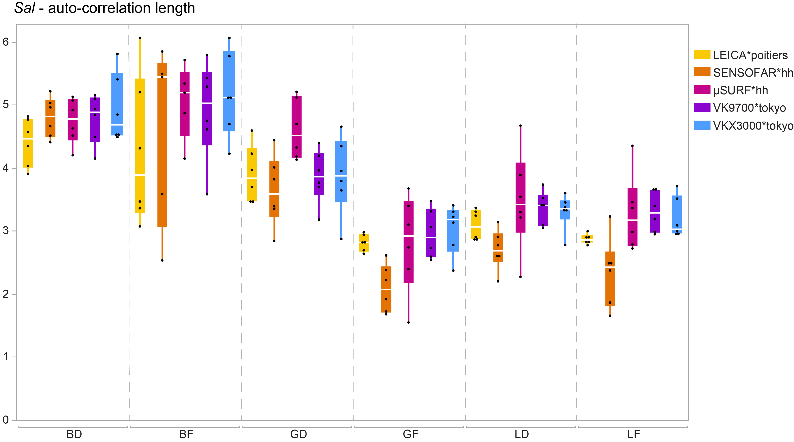* | | | | *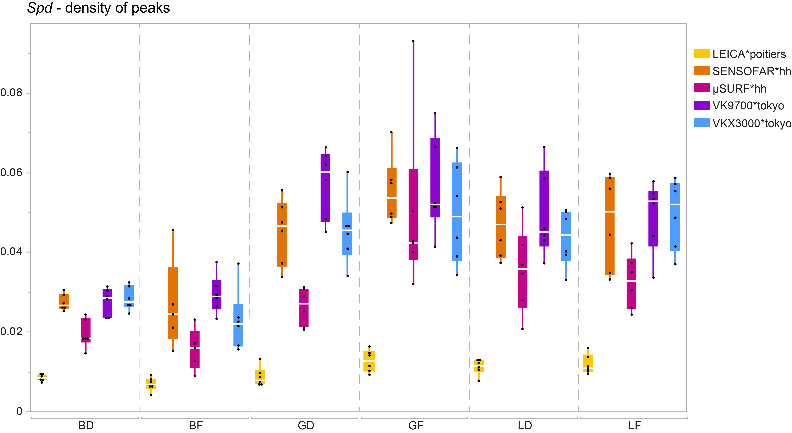* | | | *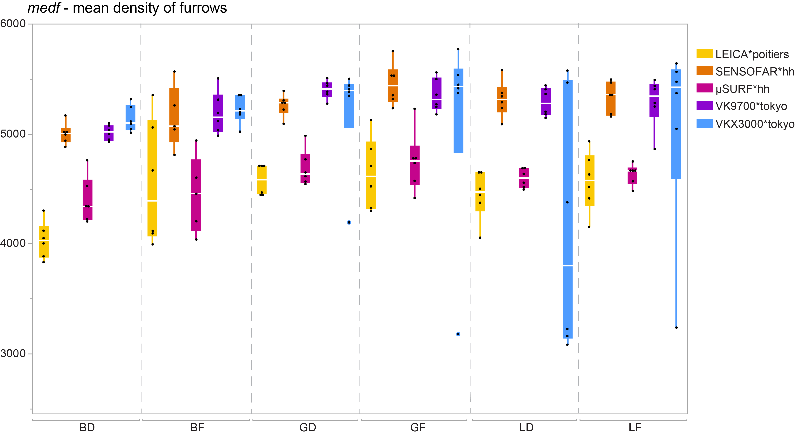* | |
| **Direction** | | | | | | | | |
| *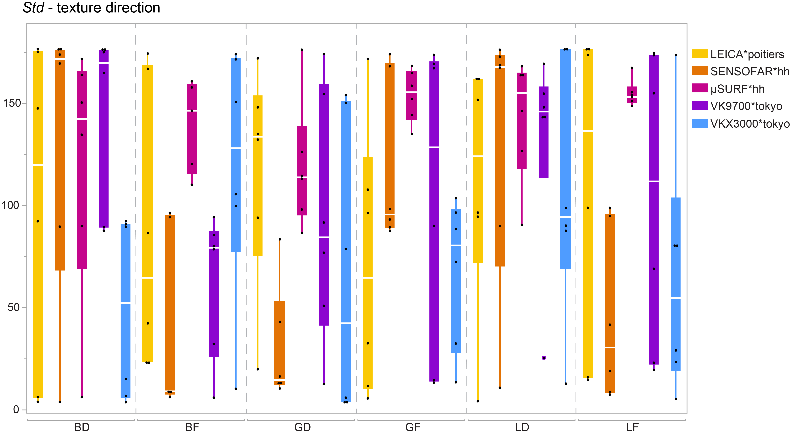* | | | | *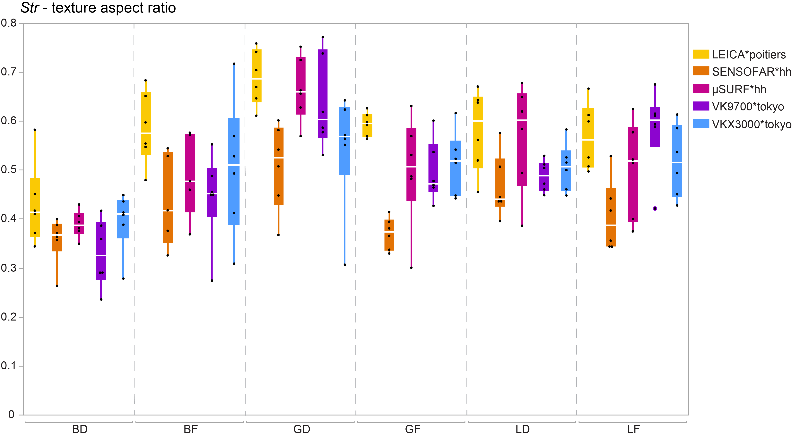* | | | | |
| *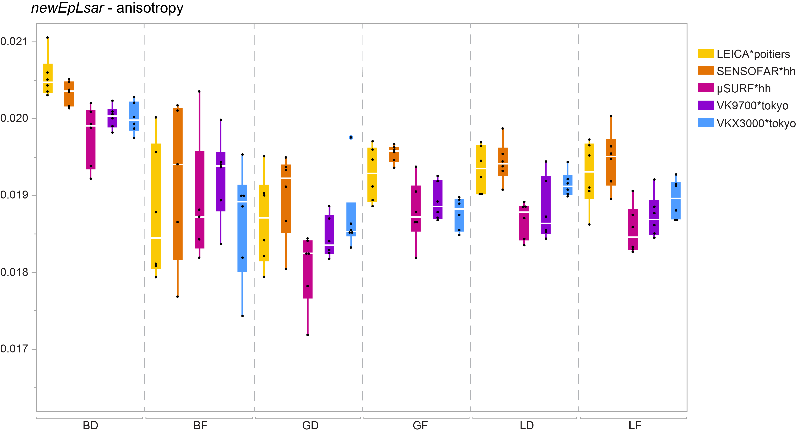* | | | | *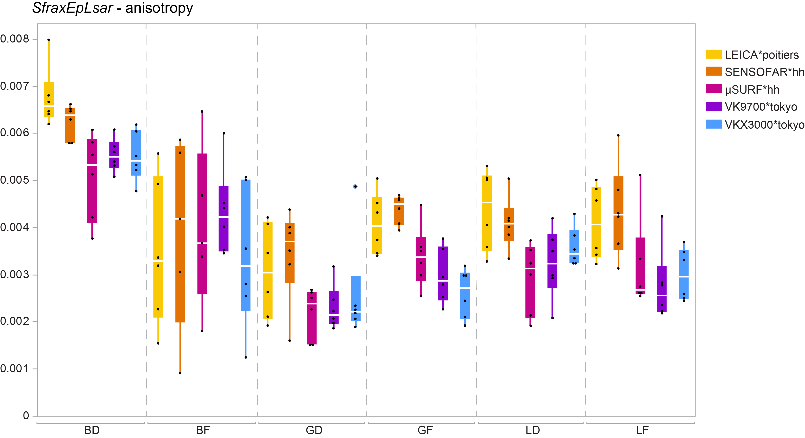* | | | | |
| **Height** | | | | | | | |  |
| *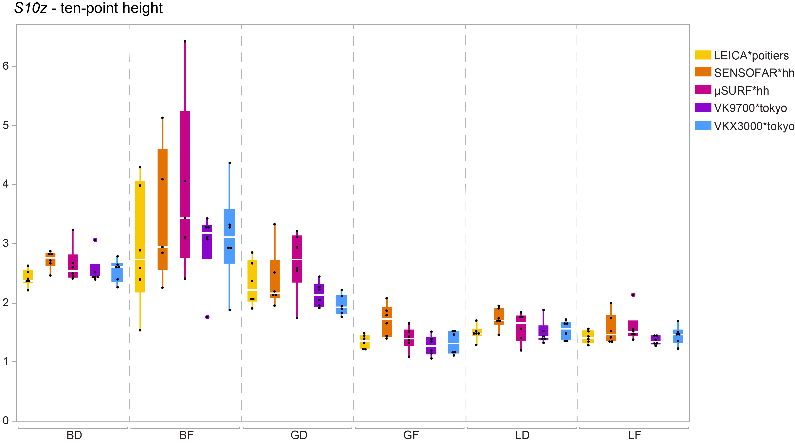* | | | *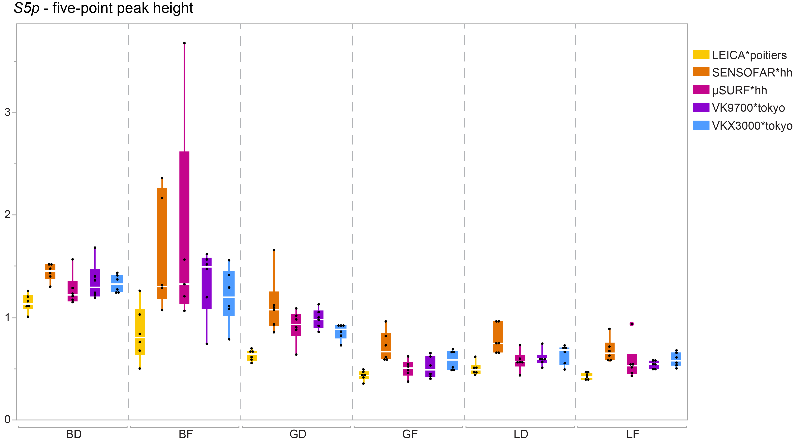* | | *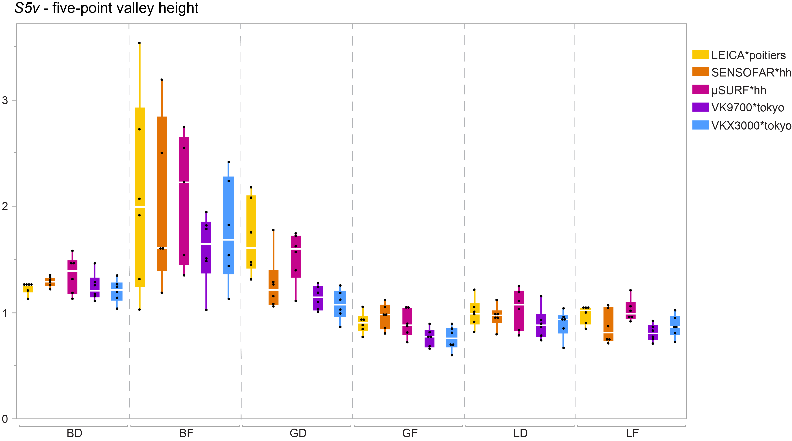* | | |  |
| *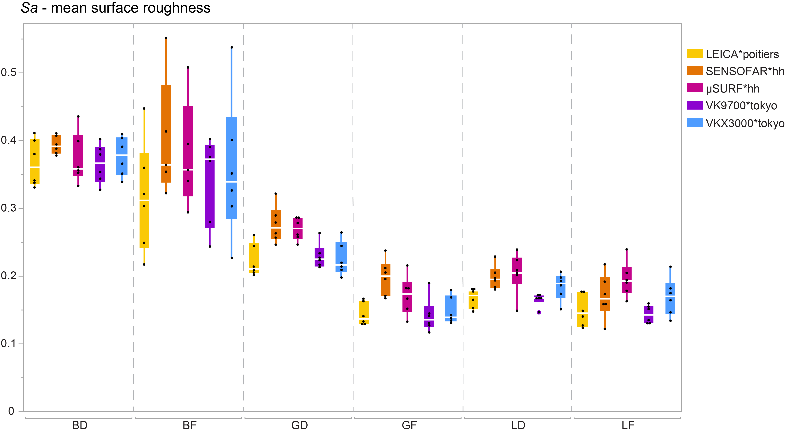* | | | *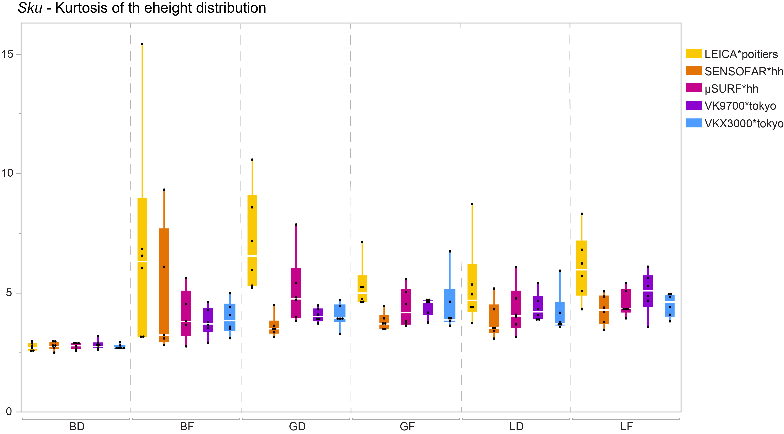* | | *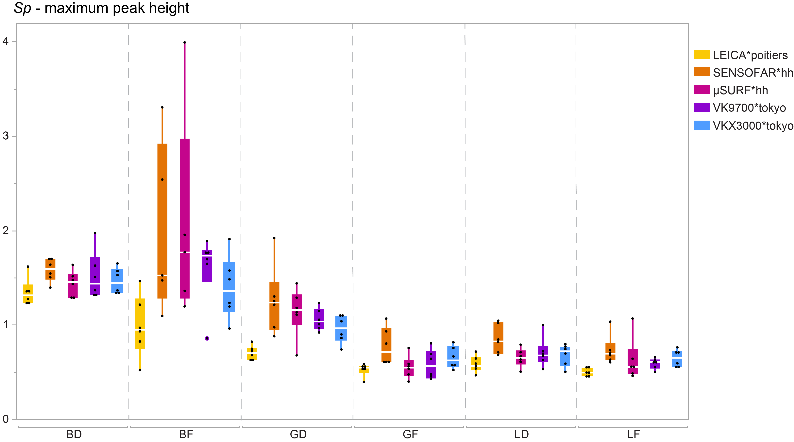* | | |  |
| *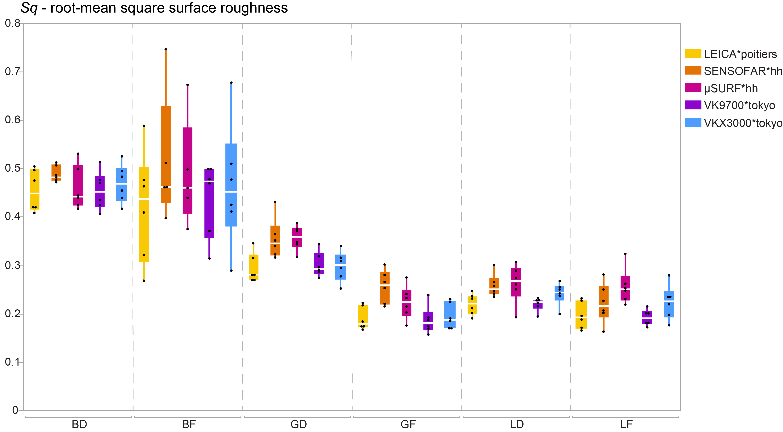* | | | *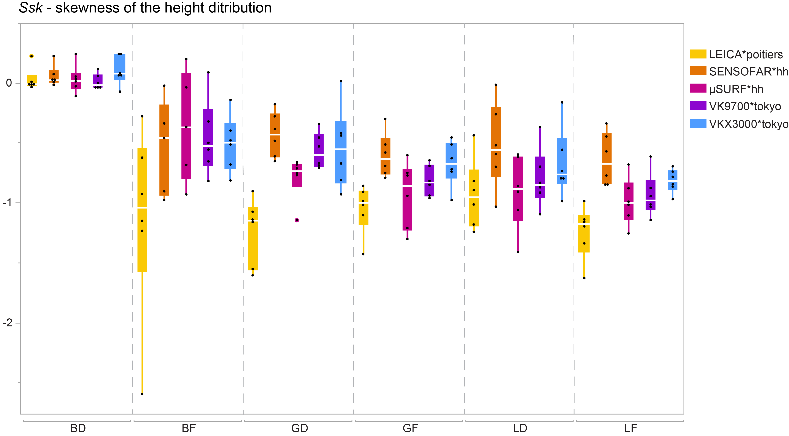* | | | | |  |
| **Height (cont.)** | | | | | | | |  |
| *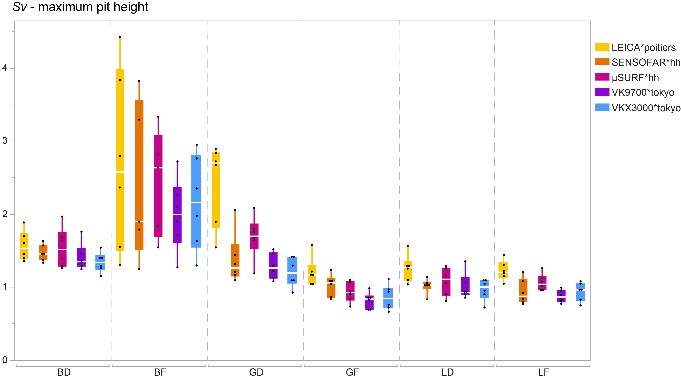* | | | *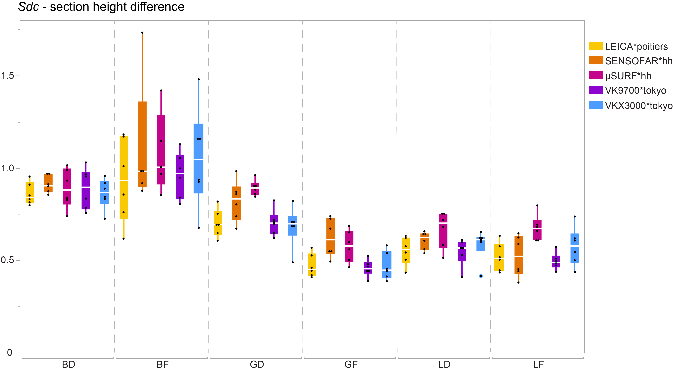* | | 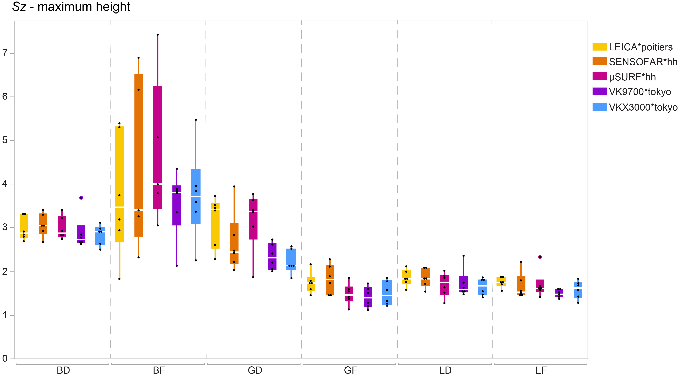 | | |  |
| *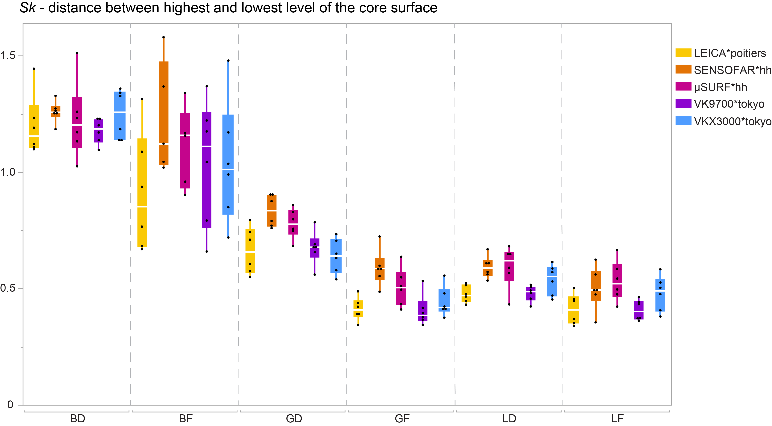* | | | *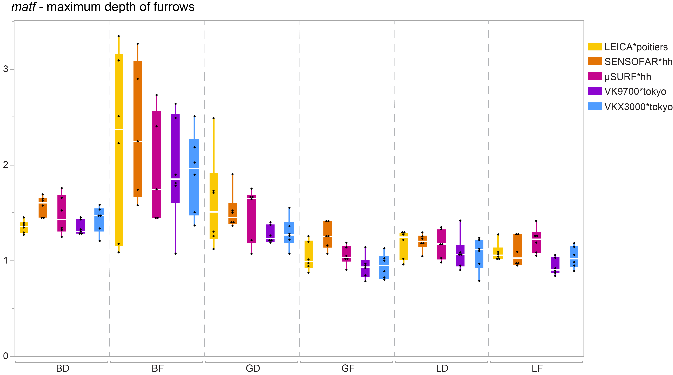* | | *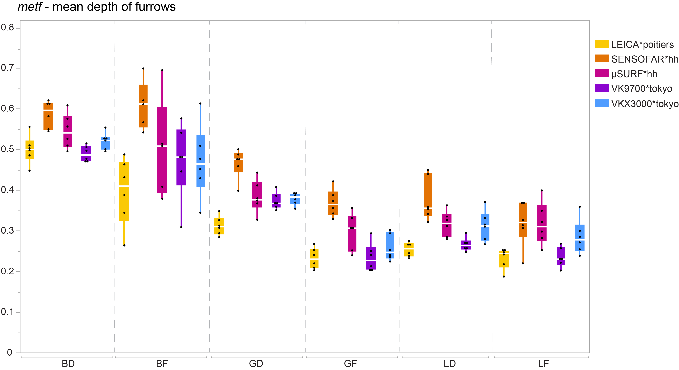* | | |  |
| *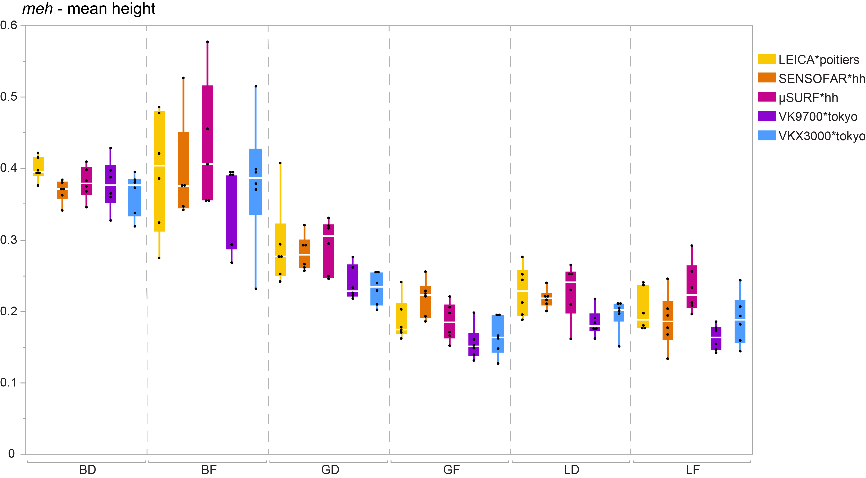* | | | | | | | |  |
| **Peak sharpness** | | **Plateau size** | | | | | |  |
| *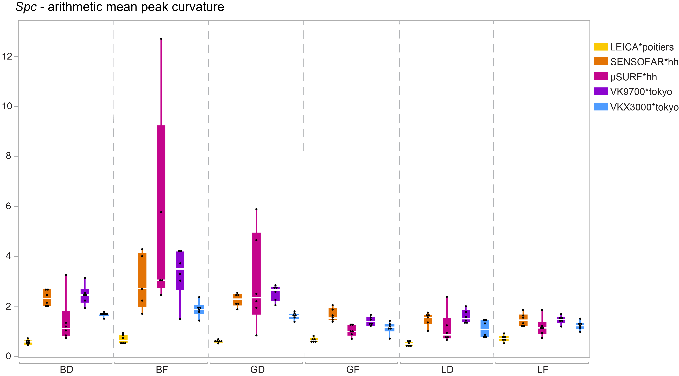* | | *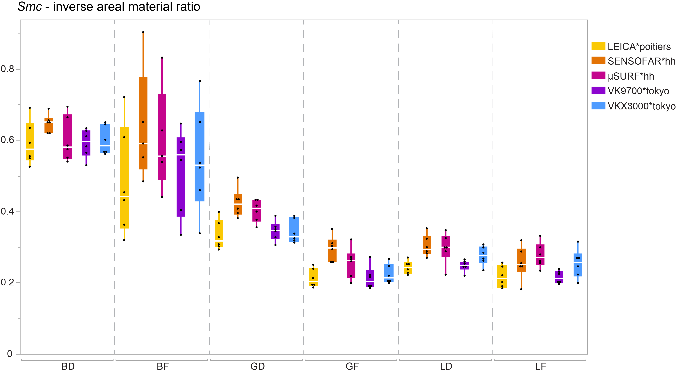* | | | | *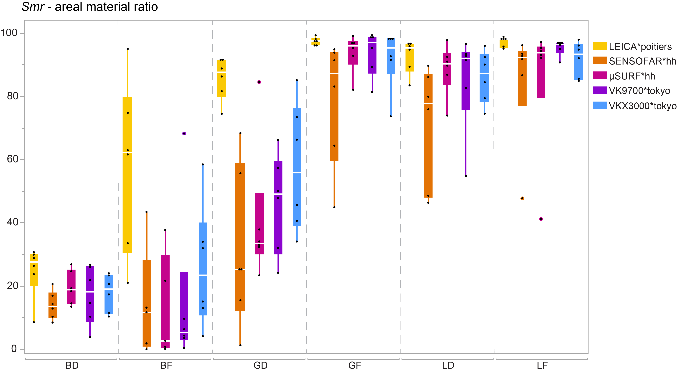* | |  |
| **Slope** | | | | | |  | |  |
| 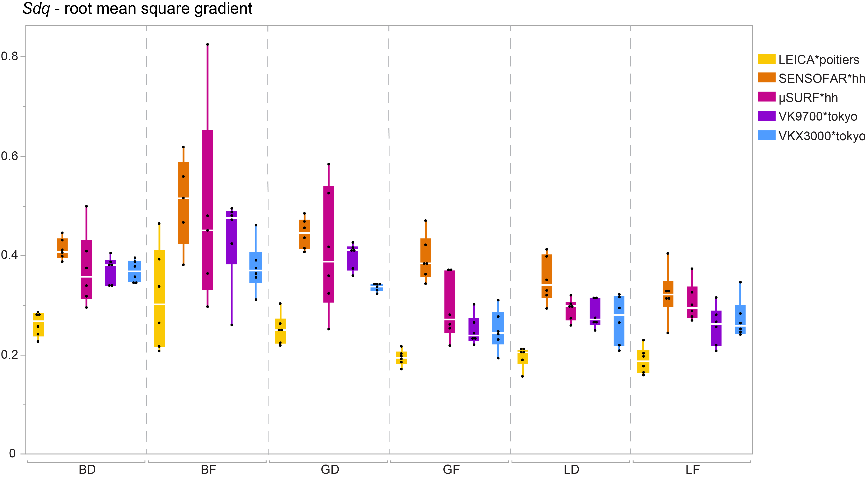 | | | | | | | |  |
| **Volume** | | | | | | | |  |
| *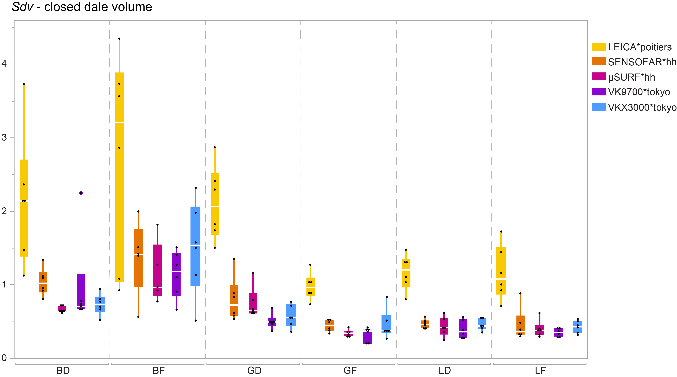* | *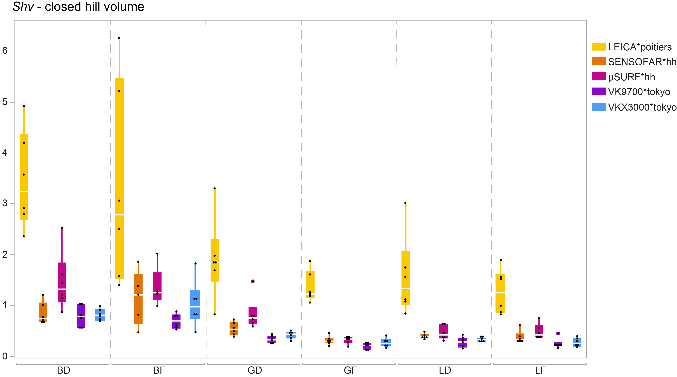* | | | | *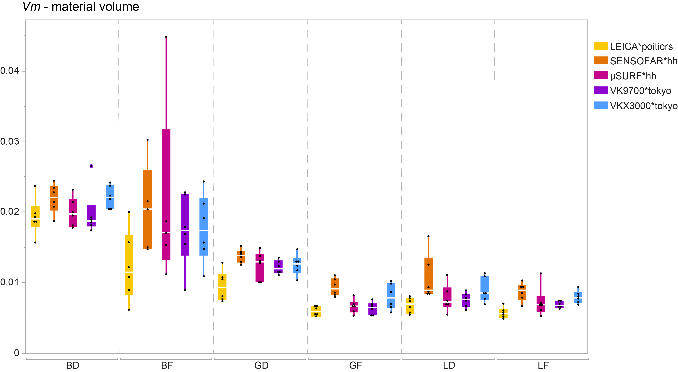* | | |  |
| *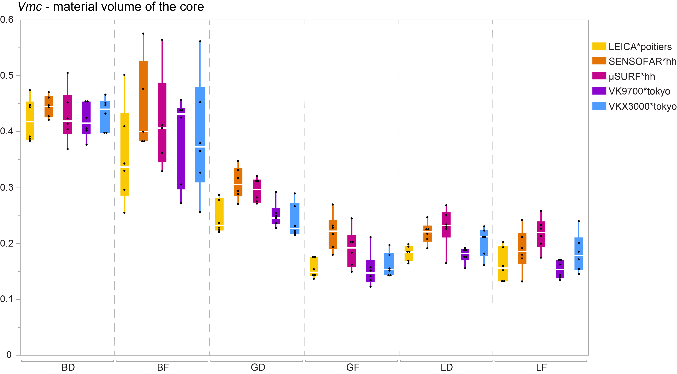* | *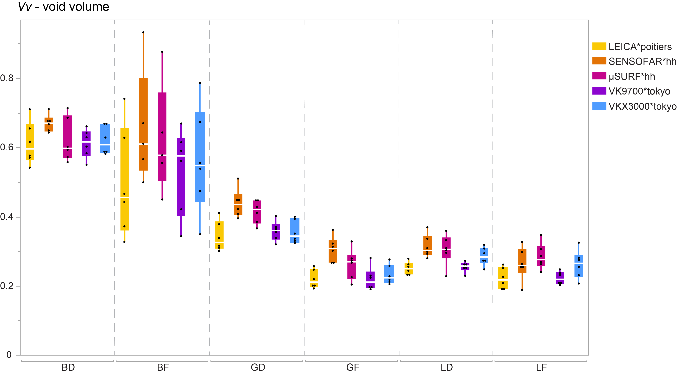* | | | | *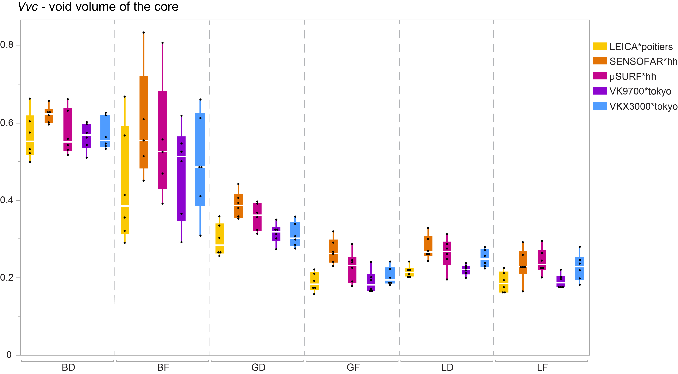* | | |  |
| *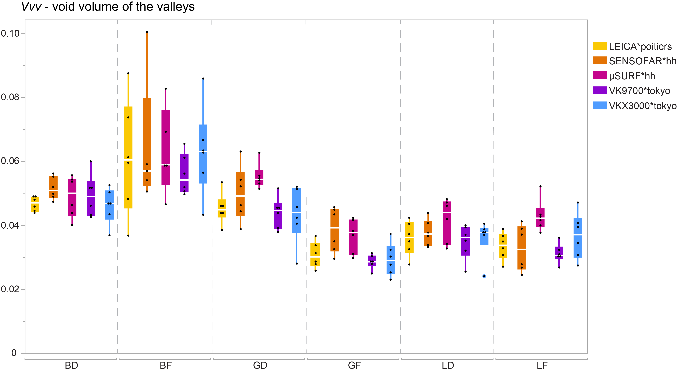* | | | | | | | |  |

**Figure S1.** **Boxplots for all 40 analyzed dental microwear texture parameters for the guinea pig dataset, measured on the five different instruments.** The thick horizontal bar represents the median; the box encloses the first (25%) and third (75%) quartiles; the whiskers extend to the full interquartile range. Abbreviations of diets groups: BD = bamboo dry, BF = bamboo fresh, GD = grass dry, GF = grass fresh, LD = lucerne dry, LF = lucerne fresh. For parameter descriptions, see Table S3

| **Area** | |
| --- | --- |
| *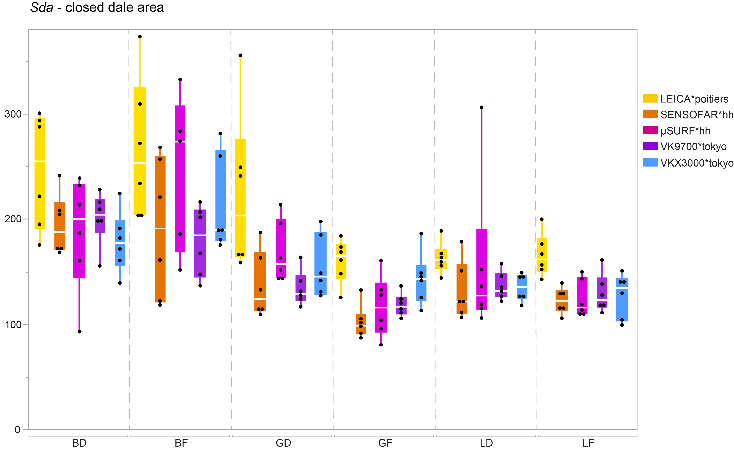* | *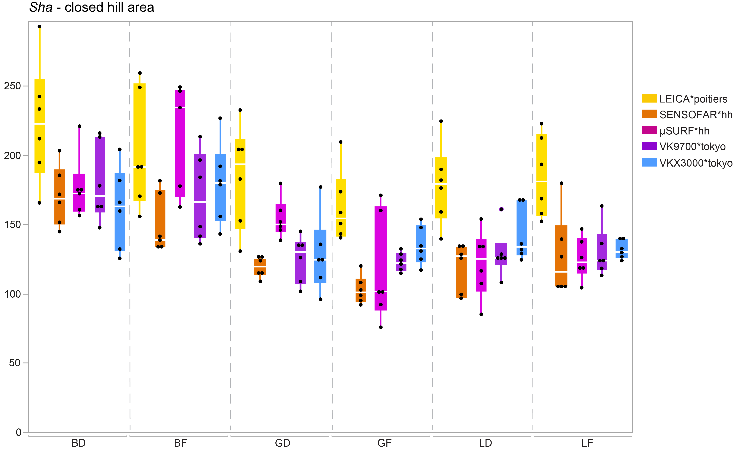* |
| *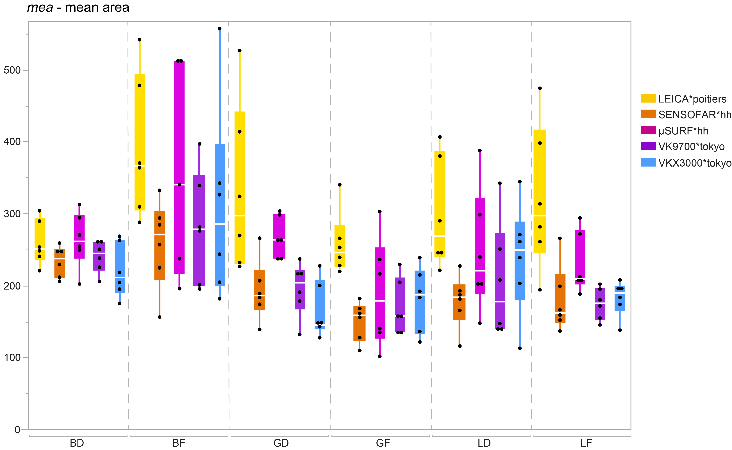* | |

| **Complexity** | | |
| --- | --- | --- |
| *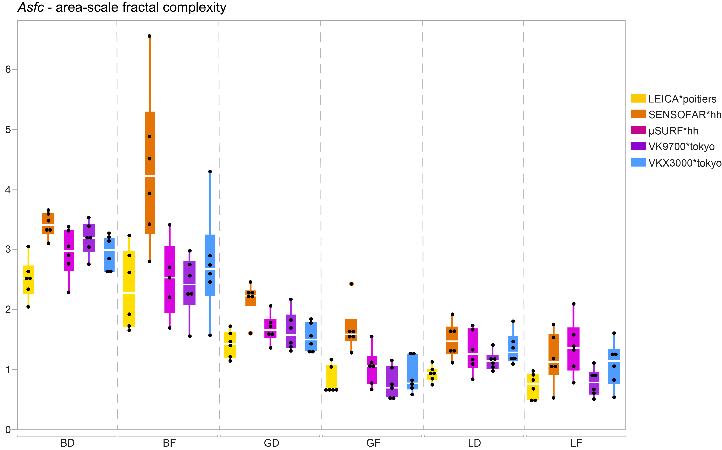* | *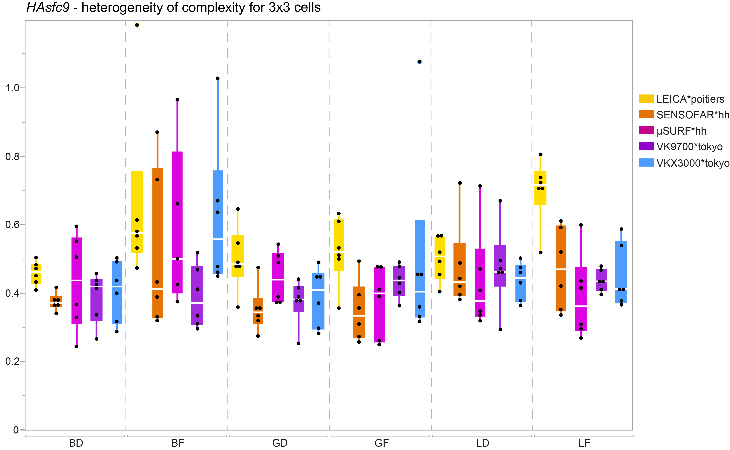* | *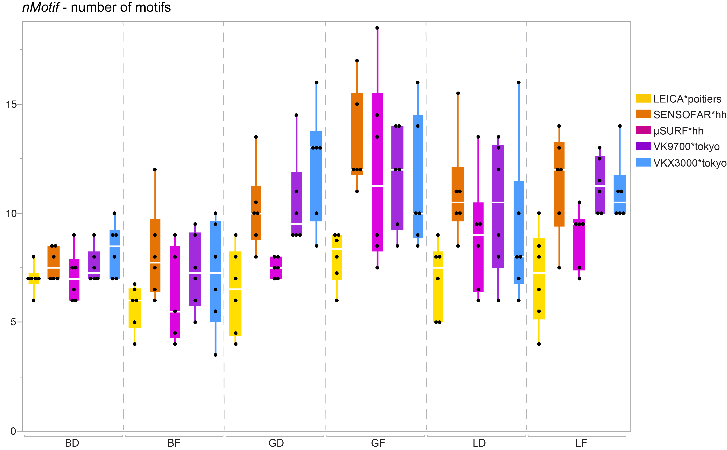* |
| *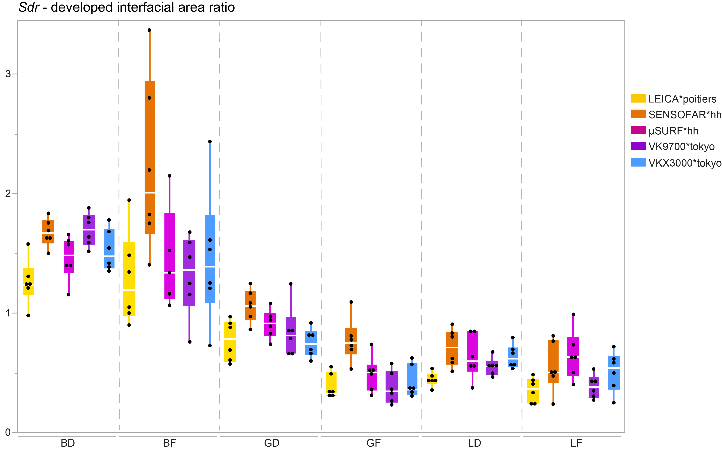* | | |

| **Density** | | | | | | | | |
| --- | --- | --- | --- | --- | --- | --- | --- | --- |
| *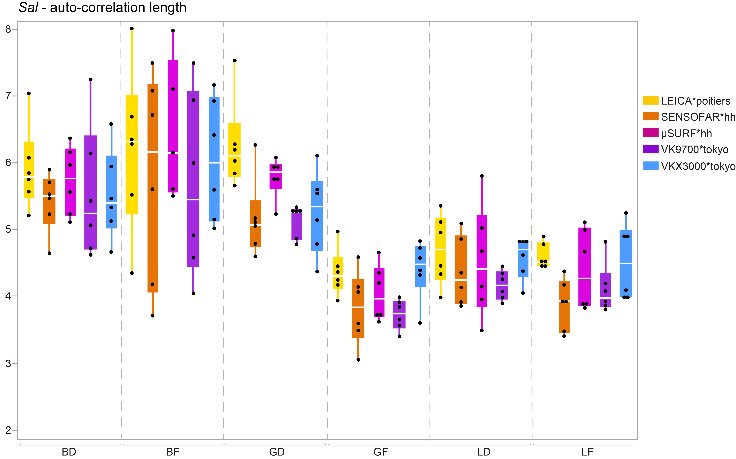* | | | | *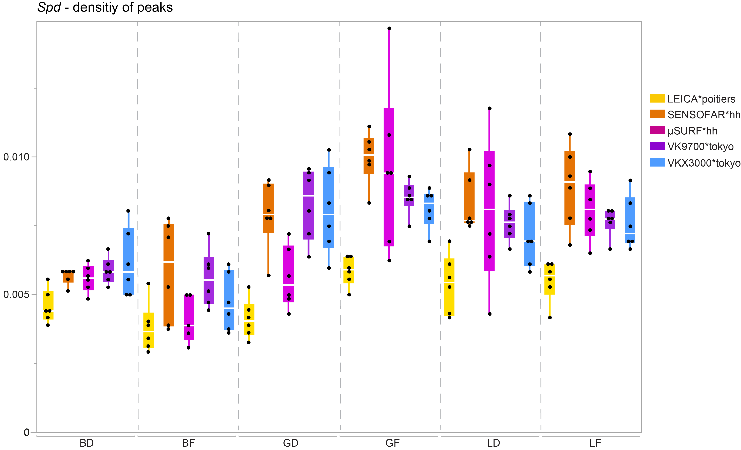* | | | *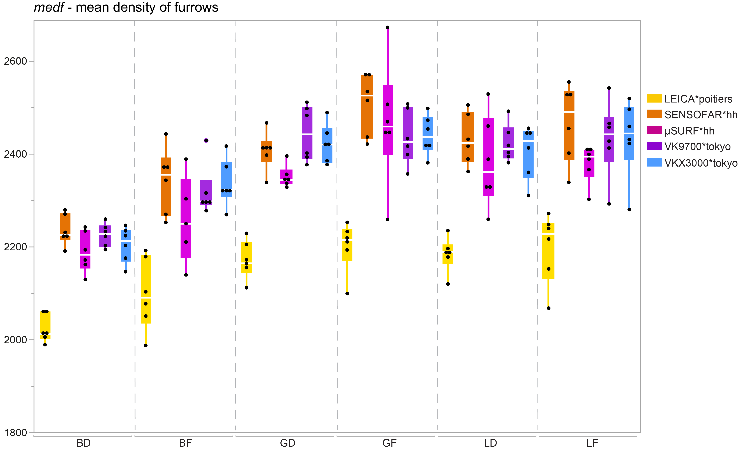* | |
| **Direction** | | | | | | | | |
| *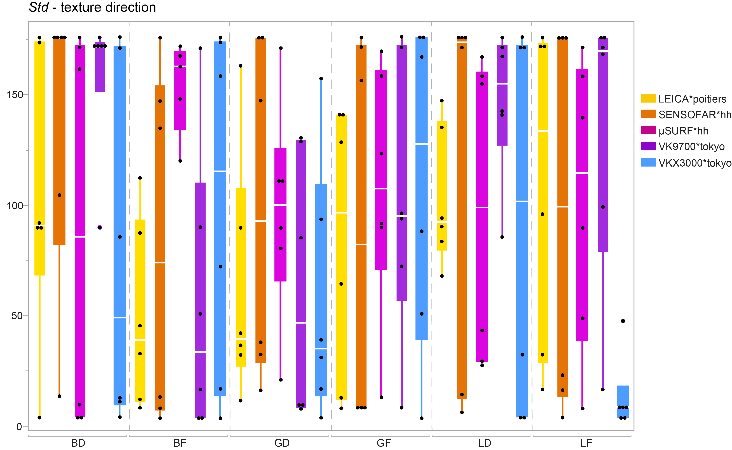* | | | | *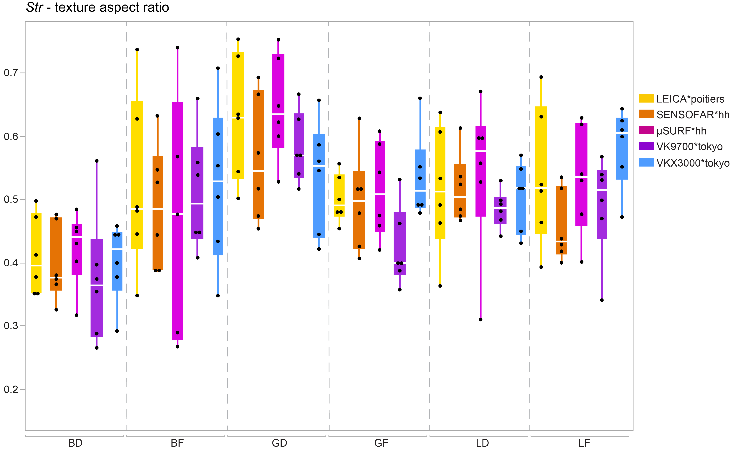* | | | | |
| *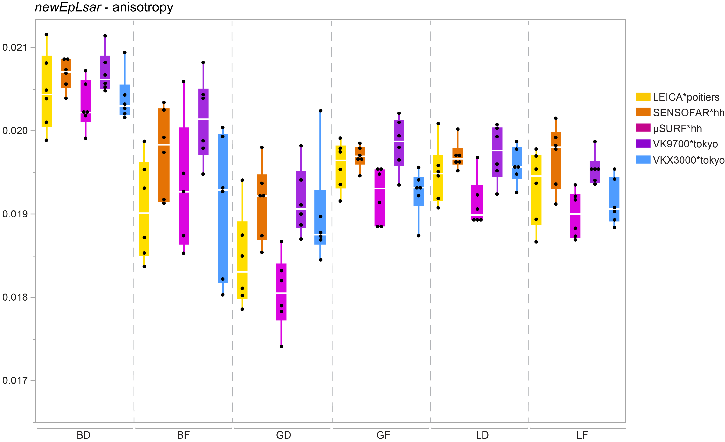* | | | | 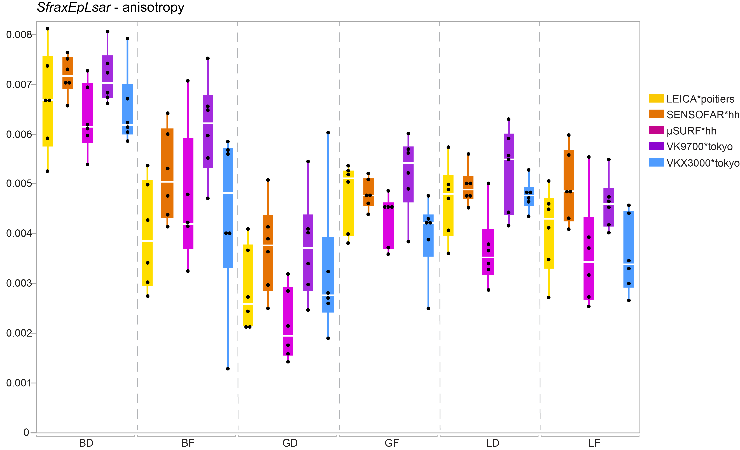 | | | | |
| **Height** | | | | | | | |  |
| *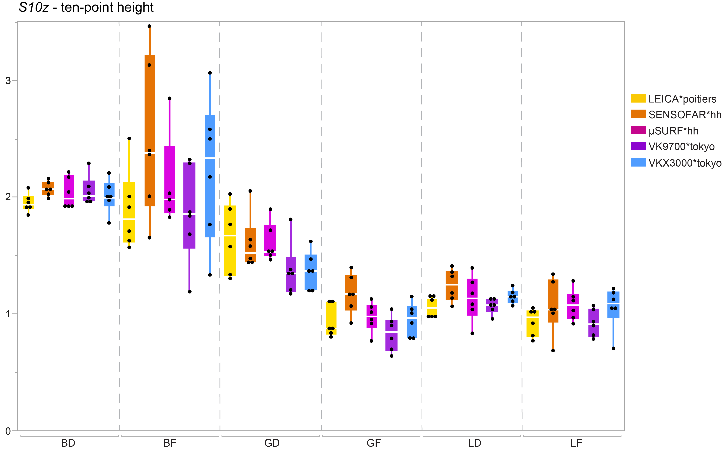* | | | *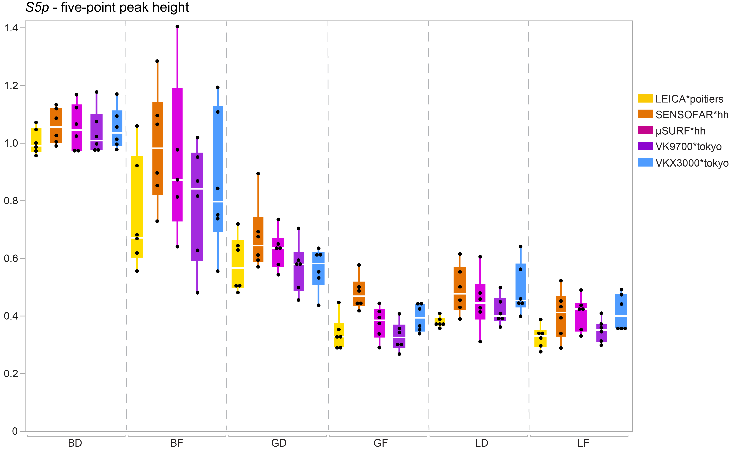* | | *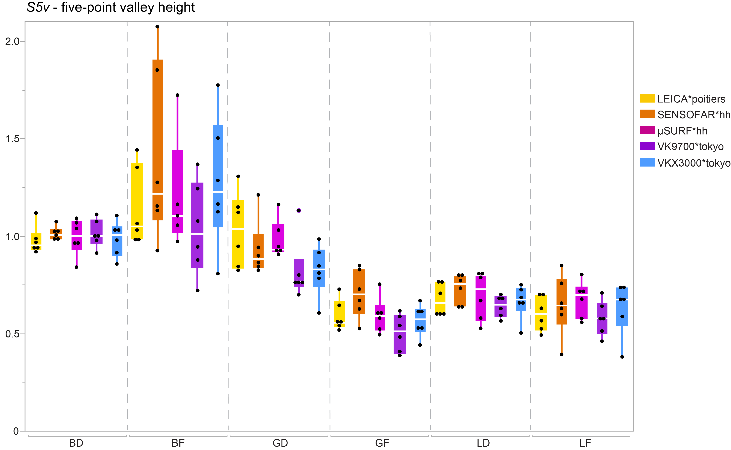* | | |  |
| *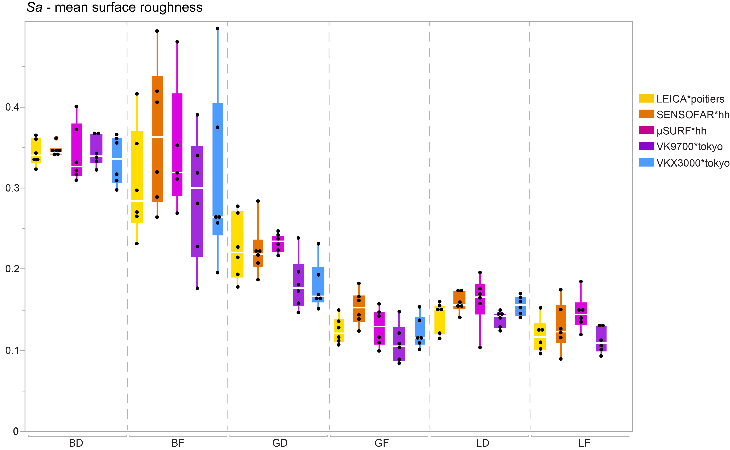* | | | *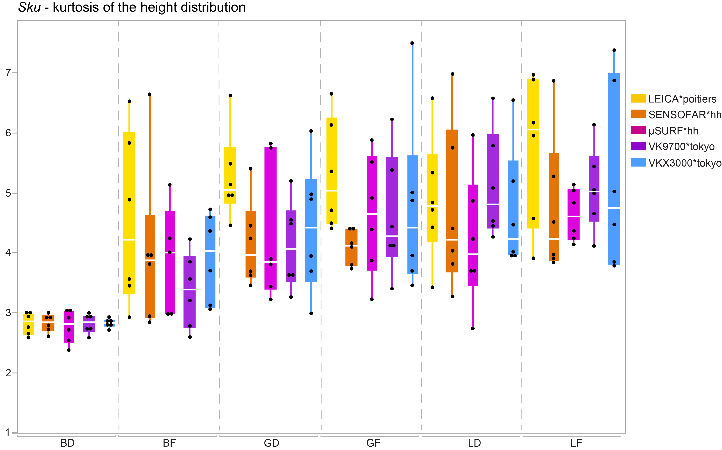* | | *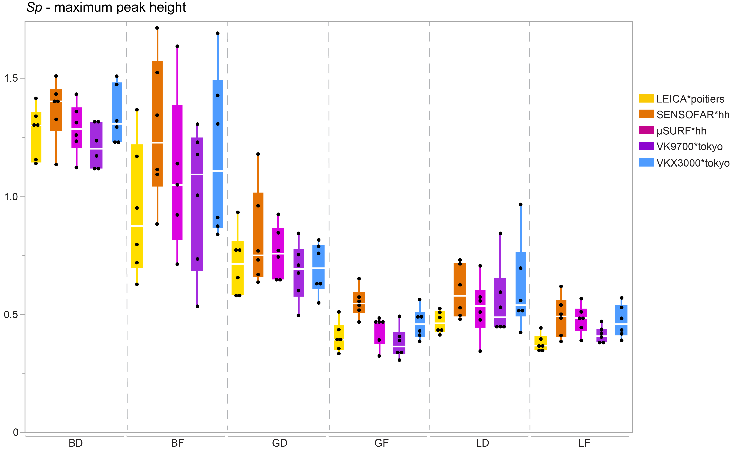* | | |  |
| *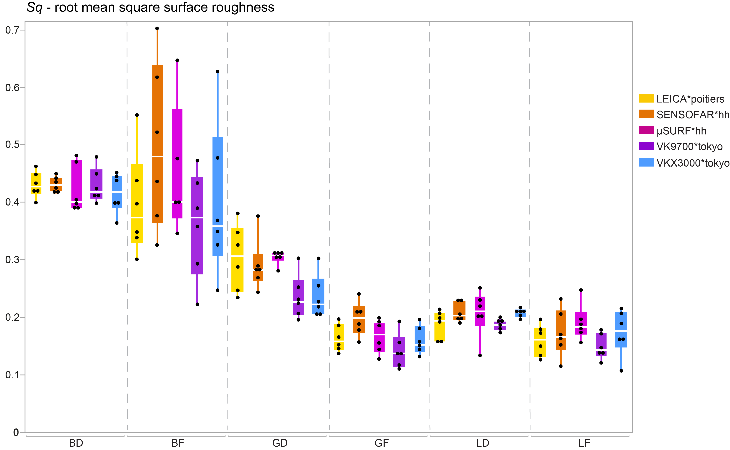* | | | *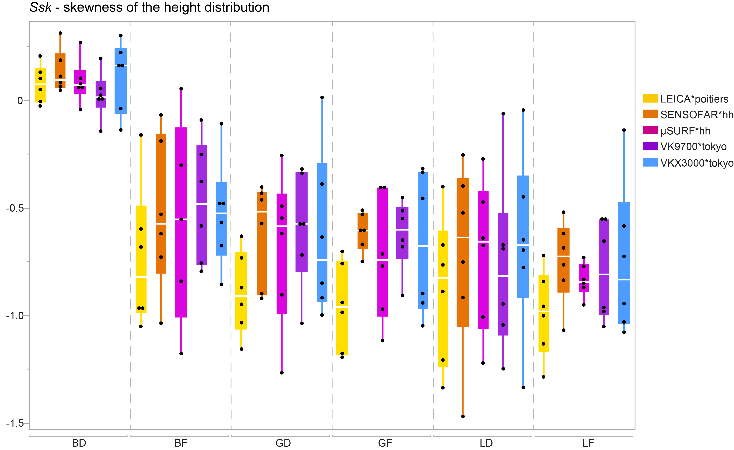* | | | | |  |
| **Height (cont.)** | | | | | | | |  |
| *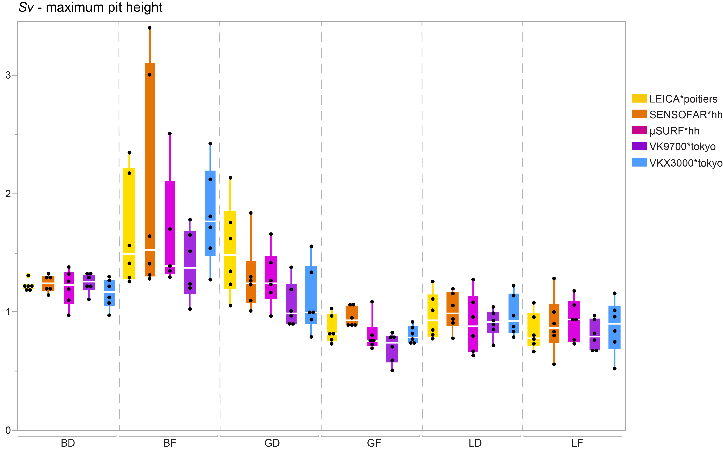* | | | *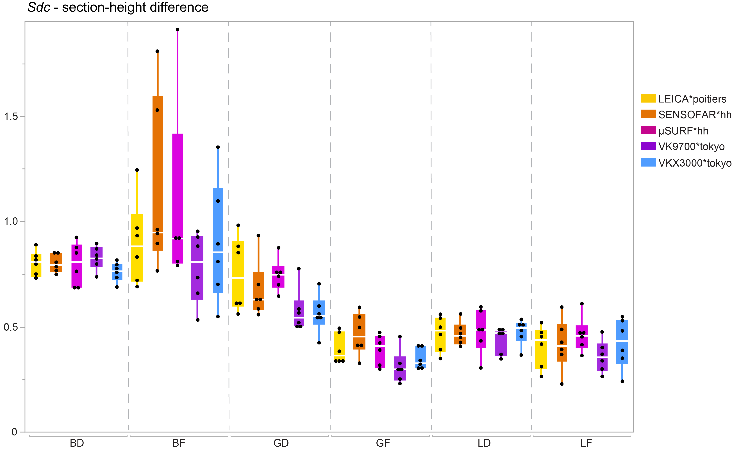* | | 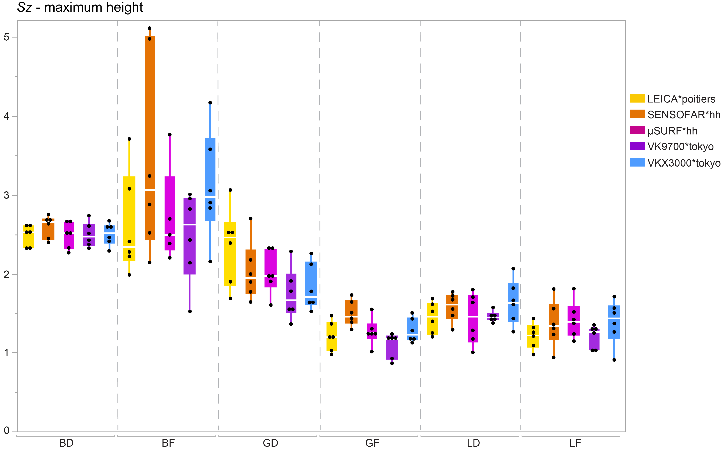 | | |  |
| *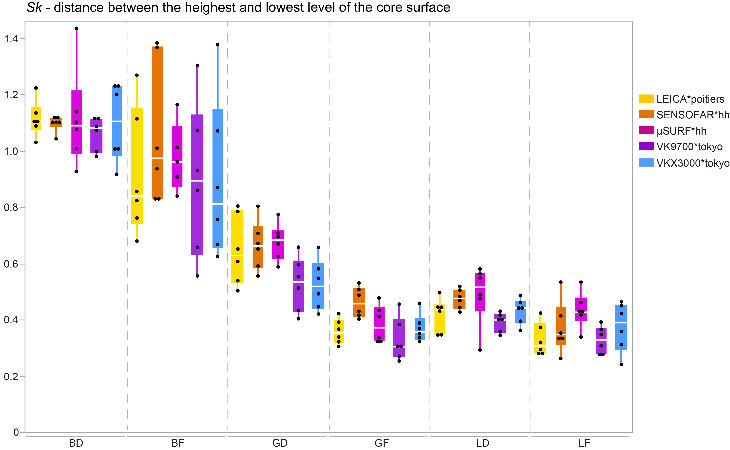* | | | *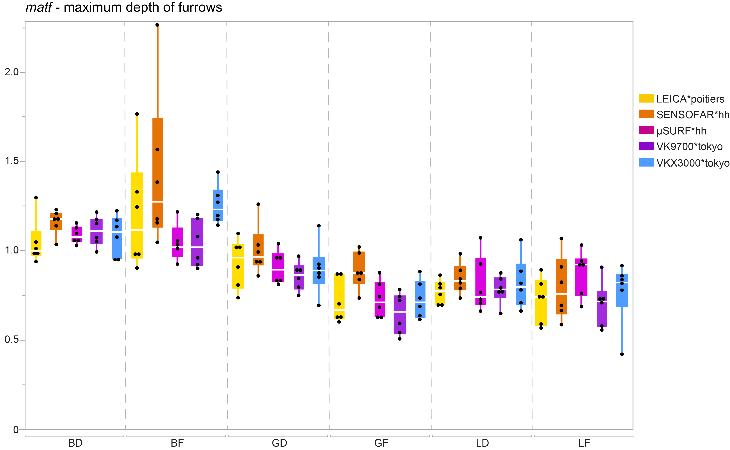* | | *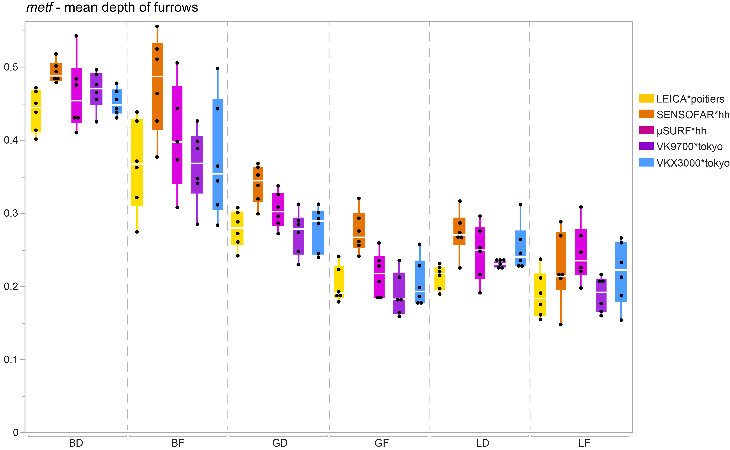* | | |  |
|  | | | | | | | |  |
| **Peak sharpness** | | **Plateau size** | | | | | |  |
| *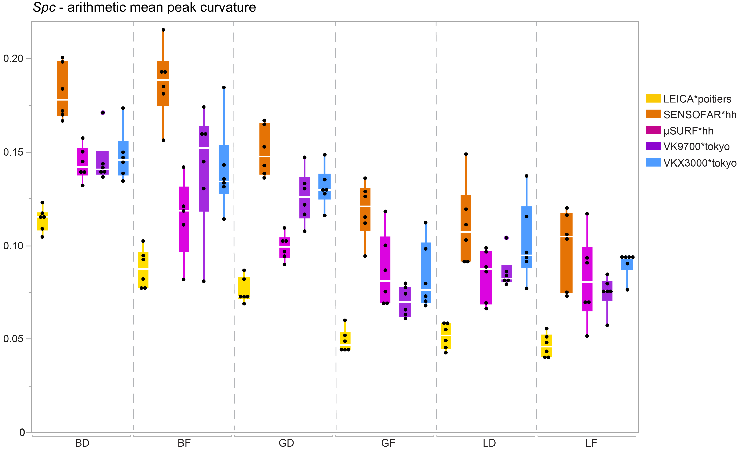* | | *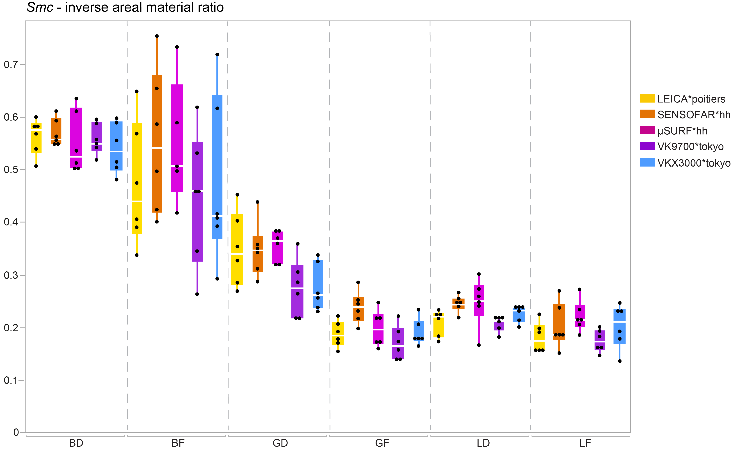* | | | | *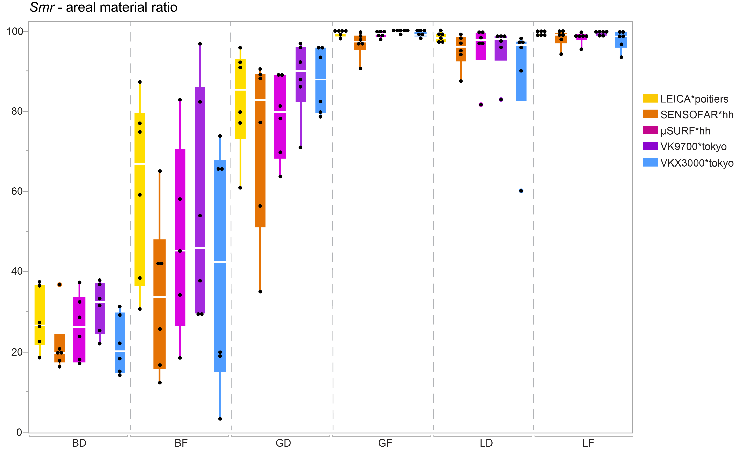* | |  |
| **Slope** | | | | | |  | |  |
| 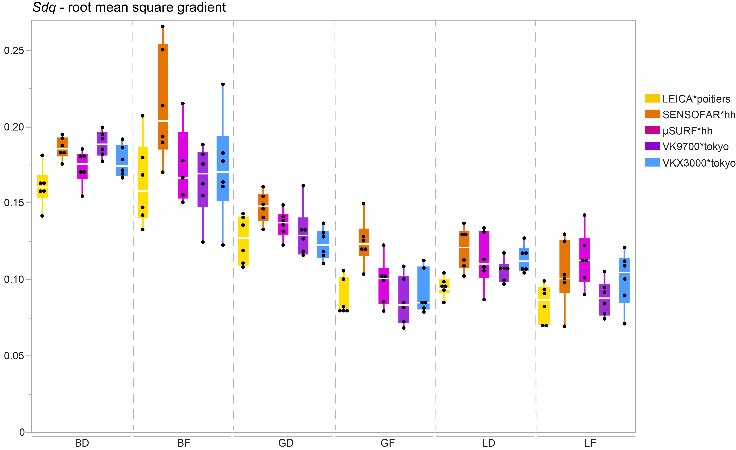 | | | | | | | |  |
| **Volume** | | | | | | | |  |
| *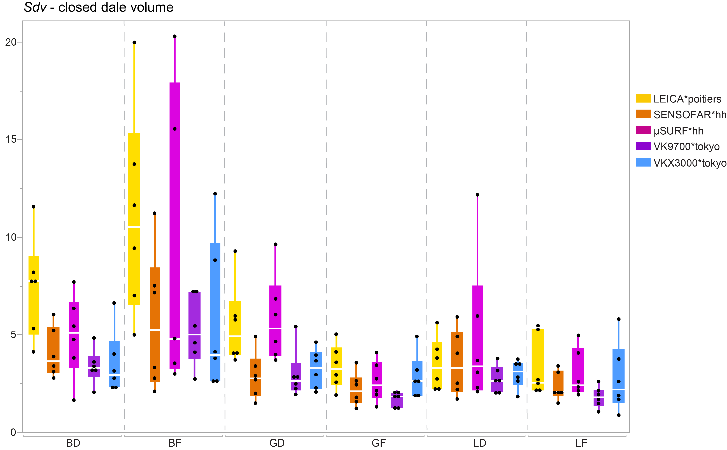* | *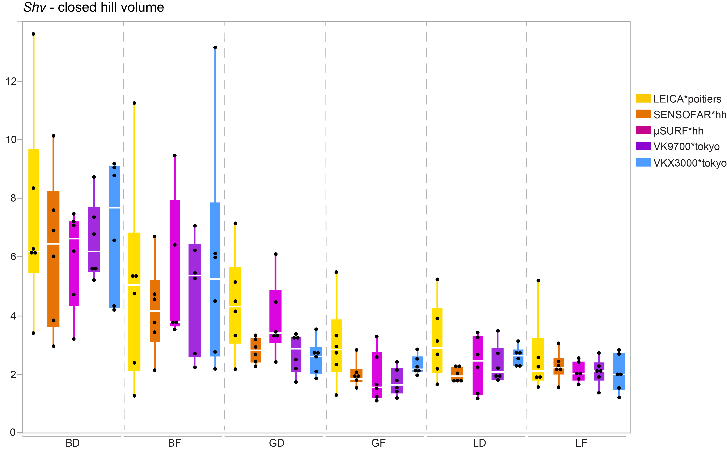* | | | | *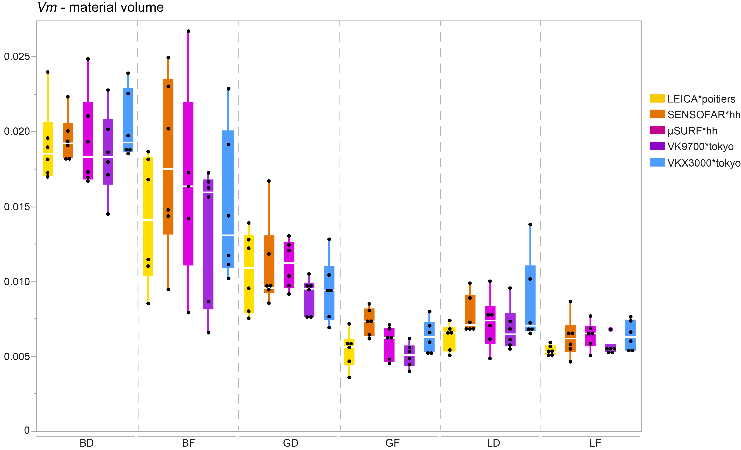* | | |  |
| *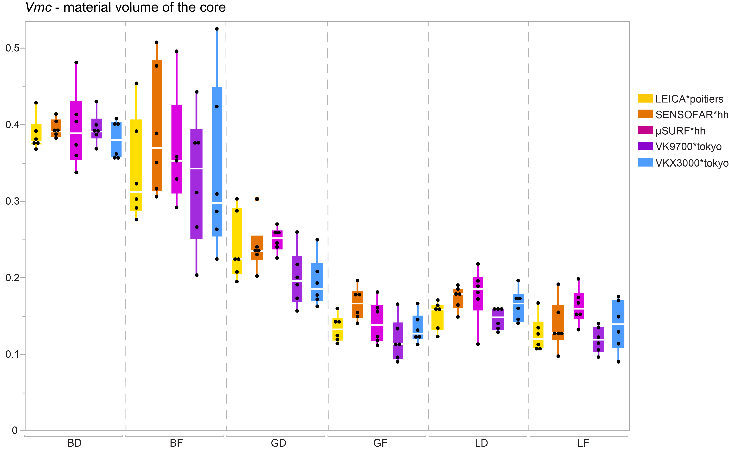* | *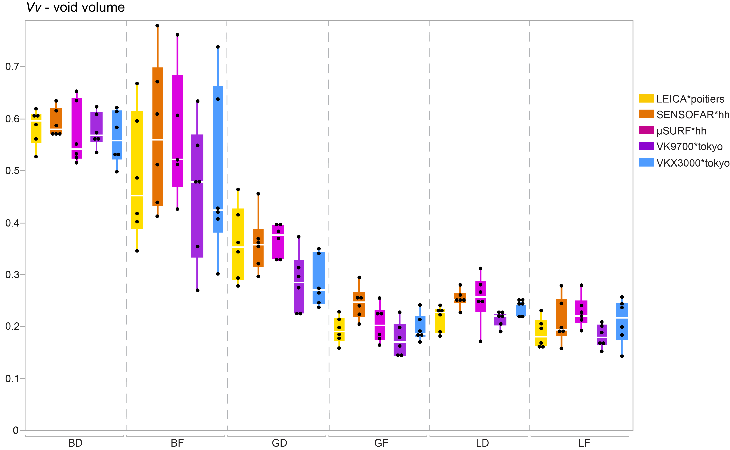* | | | | *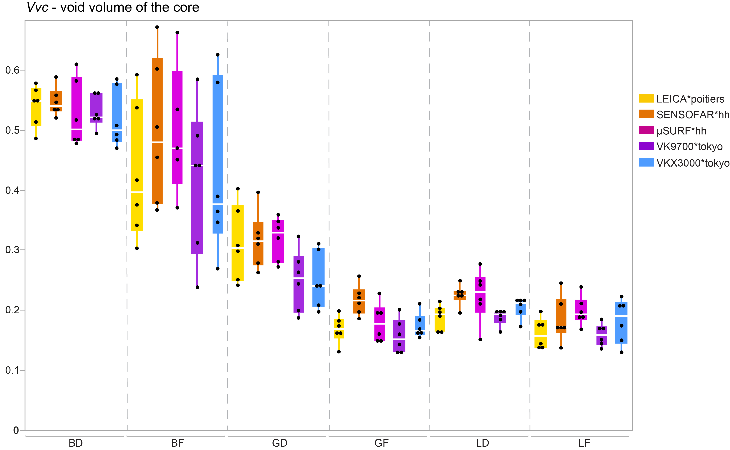* | | |  |
| 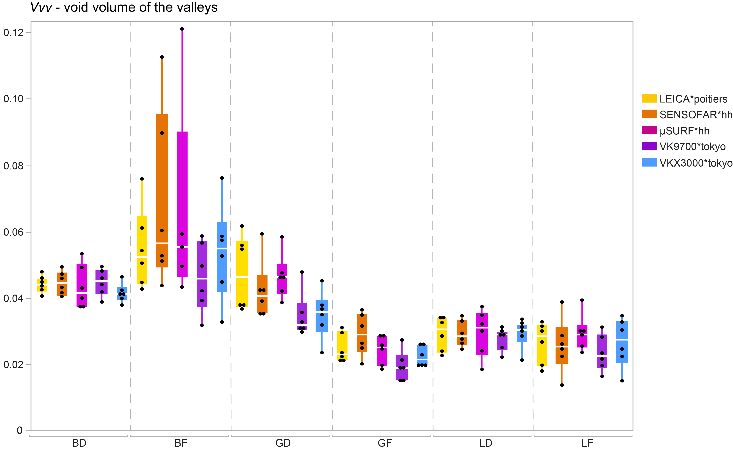 | | | | | | | |  |

**Figure S2.** **Boxplots for all 40 analyzed dental microwear texture parameters for the guinea pig dataset, measured on the five different instruments.** The thick horizontal bar represents the median; the box encloses the first (25%) and third (75%) quartiles; the whiskers extend to the full interquartile range. Abbreviations of diets groups: BD = bamboo dry, BF = bamboo fresh, GD = grass dry, GF = grass fresh, LD = lucerne dry, LF = lucerne fresh. For parameter descriptions, see Table S3

**Table S3. Dental microwear texture parameter descriptions.** Standard and units according to ISO 25178, the revised ISO 25178-2, furrow, texture direction, and scale-sensitive fractal analysis (SSFA). Functional group has been assigned by the authors for easier reference to similar parameters.

| **Parameter** | **Description (condition)** | **Standard** | **Functional group** | **Unit** |
| --- | --- | --- | --- | --- |
| *mea* | Mean area | Motif | Area | µm^2^ |
| *Sda* | Closed dale area | ISO 25178 | Area | µm^2^ |
| *Sha* | Closed hill area | ISO 25178 | Area | µm^2^ |
| *Sdr* | Developed interfacial area ratio | ISO 25178 | Complexity | % |
| *Asfc* | Area-scale functional complexity | SSFA | Complexity | no unit |
| *HAsfc9* | Heterogeneity of functional complexity | SSFA | Complexity | no unit |
| *nMotif* | Number of motifs | Motif | Complexity | no unit |
| *Sal* | Auto-correlation length (*s* = 0.2) | ISO 25178 | Density | µm |
| *Spd* | Density of peaks | ISO 25178 | Density | 1/µm^2^ |
| *medf* | Mean density of furrows | Furrow | Density | cm/cm^2^ |
| *Str* | Texture aspect ratio | ISO 25178 | Direction | no unit |
| *Std* | Texture direction | ISO 25178 | Direction | ° |
| *epLsar* | Anisotropy | SSFA | Direction | no unit |
| *New epLsar* | Anisotropy | SSFA | Direction | no unit |
| *S10z* | Ten-point height | ISO 25178 | Height | µm |
| *S5p* | Five-point peak height | ISO 25178 | Height | µm |
| *S5*v | Five-point valley height | ISO 25178 | Height | µm |
| *Sa* | Arithmetic mean height or mean surface roughness | ISO 25178 | Height | µm |
| *Sku* | Kurtosis of the height distribution | ISO 25178 | Height | no unit |
| *Sp* | Maximum peak height, height between highest peak and mean plane | ISO 25178 | Height | µm |
| *Sq* | Standard deviation of the height distribution, or RMS surface roughness | ISO 25178 | Height | µm |
| *Ssk* | Skewness of the height distribution | ISO 25178 | Height | no unit |
| *Sv* | Maximum pit height, depth between the mean plane and the deepest valley | ISO 25178 | Height | µm |
| *Sdc (=Sxp)* | Section height difference | ISO 25178-2 | Height | µm |
| *Sz* | Maximum height, height between the highest peak and the deepest valley | ISO 25178 | Height | µm |
| *Sk* | Distance between the highest and lowest level of the core surface | ISO 25178 | Height | µm |
| *meh* | Mean height | Motif | Height | µm |
| *matf* | Maximum depth of furrows | Furrow | Height | µm |
| *metf* | Mean depth of furrows | Furrow | Height | µm |
| *Smc* | Inverse areal material ratio (*p* = 10%) | ISO 25178 | Plateau size | µm |
| *Smr* | Areal material ratio, bearing area at given height | ISO 25178 | Plateau size | % |
| *Sdq* | Root mean square gradient | ISO 25178 | Slope | no unit |
| *Spc* | Arithmetic mean peak curvature | ISO 25178 | Peak sharpness | 1/µm |
| *Sdv* | Closed dale volume | ISO 25178 | Volume | µm^3^ |
| *Shv* | Closed hill volume | ISO 25178 | Volume | µm^3^ |
| *Vm* | Material volume at a given material ratio (*p* = 10%) | ISO 25178 | Volume | µm ^3^/µm ^2^ |
| *Vmc* | Material volume of the core at given material ratio (*p* = 10%, *q* = 80%) | ISO 25178 | Volume | µm ^3^/µm ^2^ |
| *Vv* | Void volume at a given material ratio (*p* = 10%) | ISO 25178 | Volume | µm ^3^/µm ^2^ |
| *Vvc* | Void volume of the core (*p* = 10%, *q* = 80%) | ISO 25178 | Volume | µm ^3^/µm ^2^ |
| *Vvv* | Void volume of the valley at a given material ratio (*p* = 80%) | ISO 25178 | Volume | µm ^3^/µm ^2^ |

**Scripts for statistical tests using JMP Pro 17.0**

Paired t-test for machine comparisons (change ”DMT parameter name” upon execution)

Data: GP_all_filters_all_datasets_MOK_Median_GroupSplit_20230926.jmp (JMP data will be provided upon reasonable request to authors)

Script:

Matched Pairs(

Y(

:”DMT parameter name” Leica*poitiers, : ”DMT parameter name” sensofar*hh, : ”DMT parameter name” usurf*hh, : ”DMT parameter name” VK9700*tokyo, : ”DMT parameter name” VKX3000*tokyo

),

Reference Frame( 0 )

)

Summary results: Supplementary Tables S1.xlsx
